# Hidden Microbiota Inhabiting in Pollen Reserves of Honey Bee (*Apis mellifera*) From Amazonas Region Revealed by DNA Metabarcoding

**DOI:** 10.1111/1758-2229.70392

**Published:** 2026-07-22

**Authors:** Jessica C. Llaja, Johann E. Oyola, Jois V. Carrion, Fernando Chuquizuta, Martha S. Calderon, Danilo E. Bustamante

**Affiliations:** ^1^ Instituto de Investigación para el Desarrollo Sustentable de Ceja de Selva (INDES‐CES) Universidad Nacional Toribio Rodríguez de Mendoza Chachapoyas Amazonas Peru; ^2^ Instituto de Investigación en Ingeniería Ambiental (INAM), Facultad de Ingeniería Ambiental, Biosistemas y de la Energía (FIABE) Universidad Nacional Toribio Rodríguez de Mendoza Chachapoyas Amazonas Peru

**Keywords:** diversity, functional redundancy, *Lactobacillus*, microbial ecology, neotropical ecosystems, Peru, Tremellomycetes

## Abstract

Pollen functions as a dynamic microbial habitat and the microbes living in pollen reserves play vital roles in pollinator health and nutrition. However, the microbiota composition of honeybee pollen reserves in biodiverse Neotropical regions remains largely unknown. This study provides the first comprehensive analysis of bacterial and fungal communities in honeybee pollen reserves across six ecosystems in the Amazonas region of Peru using high‐throughput metabarcoding of the 16S rRNA gene and ITS2 markers. We found that ecosystem type is a primary driver of community structure, with bacteria and fungi responding differently to environmental changes. Despite high taxonomic heterogeneity and a limited number of shared core microbes, the main functions of these microbes were maintained, featuring enrichment of bacterial pathways involved in nutrient metabolism and saprotrophic fungal guilds. *Lactobacillus* and an unclassified Tremellomycetes fungus were dominant, yet their abundance varied with respect to floral resource diversity. The simplified Palm Swamp ecosystem showed significantly reduced microbial diversity, underscoring the vulnerability of these communities to habitat homogenization. Our results demonstrate that the pollen reserve microbiome is assembled through environmental filtering and pollinator‐mediated selection, resulting in taxonomically flexible but functionally stable communities essential for hive processes. This work provides a foundation for understanding the microbial ecology of pollen in the Amazonas region.

## Graphical abstract

DNA metabarcoding of honey bee pollen reserves from Amazonas region reveals that microbial communities are strongly shaped by their ecosystem. Despite high taxonomic turnover across different habitats, these microbiomes maintain stable, essential functions for pollinator health, though they remain highly vulnerable to habitat homogenization.

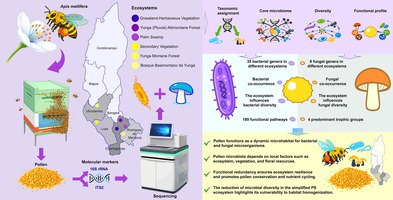

## 1 Introduction

Honey bees belong to the family Apidae and the suborder Apocrita within the order Hymenoptera (Eimanifar et al. 2018). As cosmopolitan eusocial insects, they play a critical role in sustaining plant biodiversity by pollinating diverse flora (Khan et al. 2020). They are also vital for apicultural production and global food security (Vercelli et al. 2021). However, their populations are declining because of environmental changes caused mainly by anthropogenic activities, including agricultural intensification and deforestation (Mustafa et al. 2015). This diminishes the abundance and diversity of floral resources essential for their nutrition (Crovadore et al. 2017). Honey bees host a complex microbial community, referred to as the microbiota (Compared with symbiotic microorganisms, this microbiota influences bee health, with effects ranging from beneficial to pathogenic) (Braglia et al. 2025).

The honey bee microbiome is derived from plant sources, such as pollen and nectar. Thus, an adequate supply of these resources is critical for sustaining colony productivity and ensuring long‐term survival (Braglia et al. 2025). Pollen contains specialized nonpathogenic microorganisms that play a fundamental role in metabolic functions, including biochemical and physiological processes and has bioactive properties (Anderson et al. 2023). Additionally, certain microorganisms contribute to the long‐term preservation of stored pollen by producing essential macromolecules and protecting against parasites and pathogens (Anderson et al. 2011). The pollen reserve (also known as beebread) microbiome is influenced by seasonal variations, floral resource availability and beekeeping practices (Degrandi‐Hoffman et al. 2021). Drastic changes in these factors can disrupt microbiome communities, increasing colony susceptibility or even leading to collapse (Dharampal et al. 2019). The most prevalent microorganisms associated with honey bees bread include bacteria (e.g., *Acinetobacter, Bifidobacterium, Frischella, Gilliamella, Lactobacillus* and *Snodgrassella*) (Donkersley et al. 2018; Anderson et al. 2023) and fungi/yeasts (e.g., *Aspergillus*, *Cladosporium*, *Curvularia*, *Eupenicillium*, *Fusarium*, *Gibberella*, *Mucor*, *Penicillium*, *Pestalotiopsis* and *Rhizopus*) (Disayathanoowat et al. 2020; Khan et al. 2020).

Most related research has focused on the microbiota of the honey bee intestinal tract (Engel et al. 2012). The results of genomic and metabolic studies revealed that the gut of the honeybee, which closely interacts with its microbiota, can digest and metabolize diverse plant‐derived carbohydrates (Zheng et al. 2017). Other studies have indirectly assessed the role of the honeybee microbiome in pathogen resistance, particularly concerning gut microbial communities (Guo et al. 2015; Lee et al. 2015; Maes et al. 2016). However, in the Amazonas region, no studies have characterized microbial communities in beebread (Miłek et al. 2023). Despite this limitation, microbial communities of pollen reserves differ from that of the bee gut, suggesting that these communities perform distinct functions in their respective microhabitats (Dharampal et al. 2019).

Recent advances in molecular technologies have significantly enhanced our ability to identify diverse microorganisms within specific environments. Among these methods, metabarcoding has emerged as a powerful tool for generating high‐throughput data that enable comprehensive microbial community analysis (Tremblay et al. 2019; Martin et al. 2024). This approach reveals previously undetectable microbial diversity that traditional methods cannot characterize (Manirajan et al. 2018). Metabarcoding employs high‐throughput sequencing to profile microbial communities using DNA‐based molecular markers (Martin et al. 2024). For pollen microbiome studies, the most widely used markers include the 16S rRNA gene (V3–V4 region) for bacterial identification and the ITS2 region (ITS3F/ITS4R primers) for fungal characterization. These genetic markers provide sufficient sequence variability for reliable species differentiation (Engel et al. 2012).

Metabarcoding not only facilitates the assessment of taxonomic diversity but also elucidates the functional profile of microbial communities (Carrion et al. 2025). These functional profiles represent a critical nexus between ecosystem functioning and microbial interactions (Escalas et al. 2019), enabling researchers to identify key metabolic pathways associated with specific cellular processes (Chuquizuta et al. 2026). To analyse these functions, specialized bioinformatics tools have been developed. Programs such as PICRUSt, HUMAnN, KEGG and MetaCyc leverage sequencing data to predict metabolic capabilities in bacterial communities (Sun et al. 2024). Notably, PICRUSt2 has emerged as the most widely adopted tool because of its robust ability to infer metabolic functions from genetic markers, offering comprehensive insights into environmental metabolic pathways and their ecological roles (Douglas et al. 2020). With respect to fungal communities, FUNGuild has emerged as the most powerful tool for functional annotation because of its curated ecological guild assignments (Nguyen et al. 2016). This database is built from published evidence, reducing false predictions.

Apiculture plays a significant role worldwide, supporting sustainable rural development by increasing the revenue of local economies and benefiting ecosystems via honeybee‐mediated pollination (Fedoriak et al. 2021). However, the microbiome of honey bee food sources involved in these activities, particularly that of honey bee pollen reserves (beebread) in the Amazonas region of Peru, has not been extensively characterized. DNA metabarcoding techniques have enabled detailed characterization of the honeybee pollen microbiome, providing comprehensive insights into microbial communities and their functional activities in relation to pollen grains and environmental conditions (Manirajan et al. 2018). This study aimed to characterize the microbiome of *Apis mellifera* pollen reserves using DNA metabarcoding across six ecosystems in the Amazonas region of Peru. Specifically, the (i) alpha and beta diversity indices of the pollen reserve microbiome were evaluated and (ii) the relative enrichment of predominant metabolic pathways and ecological guilds in the honey bee pollen reserve microbiome in the Amazonas region of Peru was predicted in this study.

## 2 Materials and Methods

### 2.1 Study Area and Sampling

**FIGURE 1:**
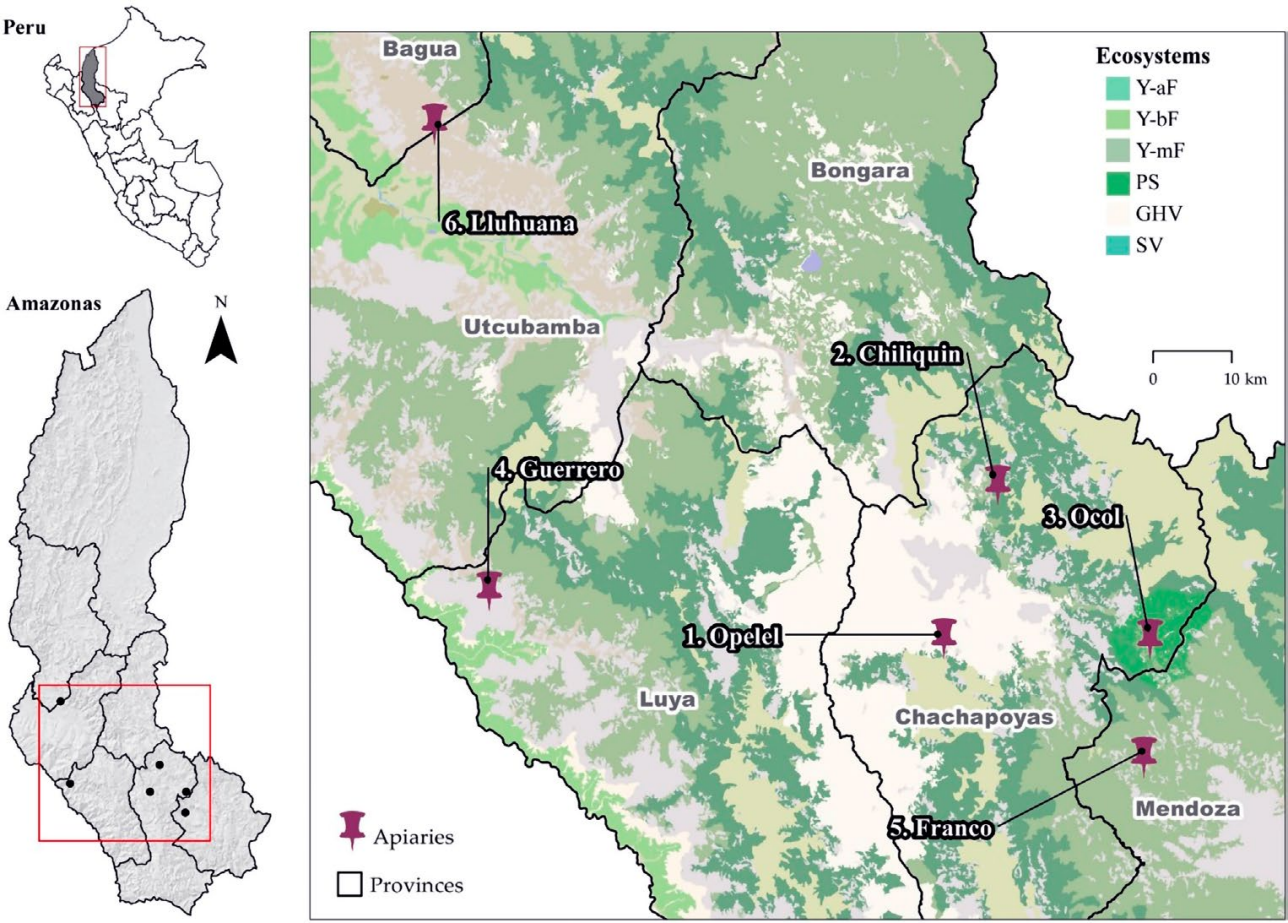
Sampling locations of the *Apis mellifera* apiaries across the six ecosystems in the Amazonas region. (1) Opelel (GHV: Grassland‐Herbaceous Vegetation), (2) Chiliquín (Y‐aF: Yunga (Pluvial) Altimontane Forest), (3) Ocol (PS: Palm Swamp), (4) Guerrero (SV: Secondary Vegetation), (5) Franco (Y‐mF: Yunga Montane Forest), (6) Lluhuana (Y‐bF: Yunga Basimontane Forest).

The apiaries included in this study were selected from the official registry maintained by the Social Development Cooperation Fund (FONCODES). The sampling sites were chosen on the basis of three criteria: (i) accessibility, (ii) vegetation type and (iii) proximity to the meteorological station. Sampling occurred during the rainy season, when precipitation levels directly influence honey bee foraging behaviour and diet (Degrandi‐Hoffman et al. 2021). Pollen reserves were collected directly from the frames 1 month after frame placement, thus representing stored rather than fresh pollen. Pollen reserve samples were collected from three hives per apiary across the following six ecosystem types in the Amazonas region: (i) the Yunga Basimontane Forest (Y‐bF), a low montane, a transitional noncloudy forest between the Amazonian lowlands and Yunga characterized by high floristic richness and tall closed‐canopy forests; (ii) the Yunga (Pluvial) Altimontane Forest (Y‐aF), a high‐elevation montane forest with steep slopes, high floristic diversity, dense epiphytic cover and patches of dwarf elfin forest at its upper limits; (iii) areas of secondary vegetation (SV), which are anthropogenically altered zones composed of fallow fields, regrowth vegetation and former pasturelands undergoing natural regeneration; (iv) areas of grassland‐herbaceous vegetation (GHV), which are open high‐Andean areas dominated by grasses and herbs, typically associated with cold climates, shallow soils and limited arboreal cover; (v) the Palm Swamp (PS), a flood‐prone Amazonian wetland dominated by dense palm stands; and (vi) the Yunga Montane Forest (Y‐mF), a humid mid‐elevation cloudy forest with abundant epiphytes, tree ferns and high structural complexity (Figure 1, Table S1). To account for honey bee flight ranges, hives within each ecosystem were spaced at least 1 km apart, following the sampling protocol of Engel et al. (2012). In brief, approximately 10 g of stored pollen from each hive was collected from interior cells using sterilized equipment. The samples were immediately placed in sterile, airtight bags, maintained in a cold chain during transport and stored at −80°C in the Molecular Biology and Genomics Laboratory of the Toribio Rodríguez de Mendoza National University until analysis. A permit for scientific research (RD‐000068‐2024‐MIDAGRI‐SERFOR‐DGGSPFFS‐DGSPF) was provided by Servicio Nacional Forestal y de Fauna Silvestre (SERFOR).

### 2.2 Sample Preprocessing

The stored pollen samples were processed by first separating the pollen grains from the wax using sterile tweezers. Approximately 3 g of the collected pollen reserves were homogenized in a sterile mortar and then transferred to a 1.5 mL microcentrifuge tube. The samples were mechanically disrupted using a BeadBug homogenizer (Benchmark Scientific, Australia), after which 750 μL of lysis buffer was added. The mixture was then incubated for 1 h at 65°C in a LabNet Digital Dry Block Heater (Model D‐1301; Edison, NJ, USA). All dissections and pollen manipulations were performed under a laminar flow hood using sterile, DNA‐free instruments. Sterile tweezers and surfaces were cleaned with 70% ethanol and UV irradiation before each sample.

### 2.3 Microbial DNA Extraction, Library Preparation and Sequencing

Microbial genomic DNA was extracted from preprocessed stored pollen samples using the ZymoBIOMICS DNA Miniprep Kit (Zymo Research, Irvine, CA, USA) according to the manufacturer's protocol. For each apiary, nine extractions were performed (three technical replicates per hive). DNA concentration and purity were measured using a NanoDrop BioSpectrometer basic (Eppendorf AG, Hamburg, Germany) and a Qubit dsDNA BR Assay kit (Invitrogen, Carlsbad CA), while DNA integrity was verified by 1% agarose gel electrophoresis. Extraction blanks (no sample) and negative controls were included throughout the DNA extraction and PCR amplification steps. No visible amplification was observed in any negative control after gel electrophoresis.

Library construction and Illumina sequencing using the MiSeq platform were performed by Macrogen (Seoul, South Korea). Genomic DNA was quantified through optical density measurements using a Qubit (Thermo Fisher Scientific, Waltham, MA, USA). DNA quality was assessed using agarose gel electrophoresis and an Agilent TapeStation. Libraries were prepared by following the Illumina Metagenomic Sequencing Library Preparation Protocol (https://support.illumina.com/documents/documentation/chemistry_documentation/16s/16s‐metagenomic‐libraryprep‐guide‐15044223‐b.pdf) in two amplification steps: an initial PCR amplification using locus‐specific PCR primers and a subsequent amplification that integrates relevant flow‐cell binding domains and unique indices (NexteraXT Index Kit, FC‐131‐1001/FC‐131‐1002). The resulting library was validated for DNA size distribution and concentration using Qubit and Tapestation instruments. To identify bacterial communities, the V3‐V4 region of the 16S rRNA gene was amplified and sequenced using the specific primers 338F (forward: 5′‐ACTCCTACGGGAGGCAGCAG‐3′) and 806R (reverse: 5′‐GGACTACHVGGGTWTCTAAT‐3′) (Claesson et al. 2009). For the fungal communities, the ITS2 gene was amplified and sequenced using the primers ITS3F (forward: 5′‐GATGAAGAACGYAGYRAA‐3′) and ITS4R (reverse: 5′‐TCCTCCGCTTATTGATATGC‐3′) (Manirajan et al. 2018). Deep metagenome amplicon sequencing was performed, generating 300 base‐paired (bp) reads each. The generated gene amplicon sequencing dataset was submitted to the NCBI Sequence Read Archive (https://www.ncbi.nlm.nih.gov/sra) under BioProject numbers PRJNA1415230 (Biosample for 16S rRNA: SAMN54926601‐SAMN54926617) and PRJNA1415238 (Biosample for ITS: SAMN54927062‐SAMN54927076).

### 2.4 Taxonomic Assignment, Core Microbiota and Diversity Analysis

The 300 bp sequence reads were trimmed as follows: (i) reverse reads were cut at 270 bp in length for bacteria and 275 bp for fungi (Phred score < 30), (ii) ambiguous or undefined bases (Ns) were not allowed and (iii) reads with a quality score < 30 were removed. The obtained sequences were processed using the ‘Dada2’ package version 1.26.0 (Callahan et al. 2016) for quality filtering and chimera removal, followed by amplicon sequence variant (ASV) inference. For taxonomic classification, the SILVA database (Release 138) (Quast et al. 2013) was used in prokaryotes through a Naive Bayes RDP classifier with 95% similarity, an 8‐size kmer and 100 bootstrap replicates, whereas the Unite v8.3 database was employed in fungi following the same parameters. The composition of the bacterial and fungal communities was presented as relative abundance data, and the most abundant microbial taxa were presented at different levels (phylum, class and genus) for each ecosystem location, following the default parameters of the ‘microViz’ package, version 0.10.08.

For the core microbiota, Venn diagrams were used to identify exclusive and shared taxa among ecosystems. Relative percentages were calculated on the basis of the total number of bacteria and fungi detected. These analyses were performed in R using the phyloseq package with rarefied data objects. Additionally, co‐occurrence analysis for bacterial and fungal datasets was conducted by integrating rarefied abundance, taxonomy and metadata tables into a microtable object. Networks were constructed at the genus level on the basis of Spearman correlations using a WGCNA approach (thresholds set at *p* < 0.01 and ∣*r*∣ ≥ 0.5). Modules were detected via the fast greedy algorithm, and relevant network attributes were calculated. Finally, the co‐occurrence networks were exported in .gexf format for visualization in Gephi, with genus‐level taxonomic information incorporated into the nodes.

The alpha diversity indices (i.e., the Shannon and Simpson indices) were calculated for each ecosystem using the diversity function of the ‘Phyloseq’ package version 1.42.0 (McMurdie and Holmes 2013). Beta diversity, structures and similarities between microbial communities at the collection sites were analysed using the Bray–Curtis dissimilarity matrix for principal coordinate analysis (PCoA) with the ‘vegan’ package version 2.6–4. All the packages used in this analysis were performed in ‘R’ software version 4.2.2.

### 2.5 Functional Profiling

The PICRUSt2 software package was used to understand the potential functional genetic capabilities of the identified bacterial communities (Douglas et al. 2020). This analysis was performed using nonrarefied data and decontaminated ASVs to maintain the integrity of the original distribution, allowing an accurate representation of the abundance of predicted gene families. These data were then rigorously filtered to identify KEGG orthology (KO) markers using a threshold of 1000 counts per million (cpm) after normalization. Gene family predictions were combined with the relative abundance of genes in the samples. In addition, a differential pathway analysis was performed using the ‘ALDEx2’ method to identify variations between the genera of the microbial communities and their respective groups (Fernandes et al. 2014). The data were visualized using ‘ggpicrust2’ version 1.7.2 in R version 4.2.2 to interpret the functional profile of the bacterial microbiota associated with pollen reserves across the six ecosystems.

The FUNGuild tool was employed to predict the ecological functions of fungal ASVs through database‐driven taxonomic annotation. Prior to annotation, a consolidated table integrating taxonomic classifications with abundance data was constructed. This table was processed using the FUNGuild command‐line utility (Guilds v1.1.py) to generate functional annotations and assign confidence levels categorized as highly probable, probable, or possible (Nguyen et al. 2016). The relative abundance of dominant fungal guilds was normalized to the total abundance of each sample and visualized via a heatmap generated in R, enabling cross‐ecosystem comparisons of fungal community functional profiles.

## 3 Results

### 3.1 Microbial DNA Sequencing of Bacteria (16S rRNA) and Fungi (ITS)

The numbers of unprocessed reads for the pollen samples obtained from the six ecosystems were 1,582,764 for 16S rRNA (Bacteria) sequencing and 2,057,083 for ITS (fungi) sequencing (Table S2). All these reads had an average length of 300 bp. After quality and filtering analysis of the forward and reverse reads, clean sequence reads with Phred score values above 30 were obtained (Figures S1 and S2, Table S1). Chimeric, plastid and mitochondrial sequences were filtered out to obtain effective sequence reads for subsequent analyses (Table S2). After normalization, the numbers of effective ASVs for the microbiota associated with pollen reserves from the six ecosystems were 533 for bacteria and 137 for fungi. After quality filtering and optimal read selection, sample 3 from the PS and samples 2 and 3 for Y‐bF ecosystem yielded zero usable reads. Consequently, these samples were excluded from all downstream analyses.

### 3.2 Taxonomic Assignment and Abundance of Bacterial Microbiota Associated With Pollen Reserves

A total of 11 bacterial phyla were identified across the six ecosystems (Figure S3, Table S3). The most abundant phyla were Firmicutes (83.81%), Proteobacteria (14.84%) and Actinobacteriota (0.73%) (Table S3). Firmicutes was the most abundant phylum in the Y‐bF (93.67%) and the Y‐aF (93.21%) ecosystems. Proteobacteria was more abundant in the PS (55.6%) and Y‐mF (18.81%) ecosystems. Actinobacteriota was primarily found in the PS ecosystem (3.42%) (Figure S3, Table S4). Several phyla with low abundance including Deinococcota (0.02%), Gemmatimonadota (0.06%) and Verrucomicrobiota (0.03%), were found exclusively in the Y‐bF ecosystem (Table S4).

Thirty‐four bacterial families were identified at the family taxonomic level. Lactobacillaceae dominated the bacterial community (82.38%), followed by Moraxellaceae (2.55%) and Beijerinckiaceae (2.39%) (Figure S4, Table S5). The abundance of Lactobacillaceae was particularly high in two ecosystems: the Y‐bF (93.28%) and Y‐aF (92.85%) (Figure S4, Table S6). Distinct distribution patterns were observed for other dominant families: the abundance of Moraxellaceae was the highest in the Y‐mF (5.78%), while the abundance of Beijerinckiaceae was the highest in the PS (15.26%) (Table S6). The PS ecosystem exhibited the greatest variability in bacterial family composition. The least abundant families were Intrasporangiaceae and Micromonosporaceae (both 0.01%), which were detected exclusively in the Y‐bF ecosystem (Figure S4, Table S6).

**FIGURE 2:**
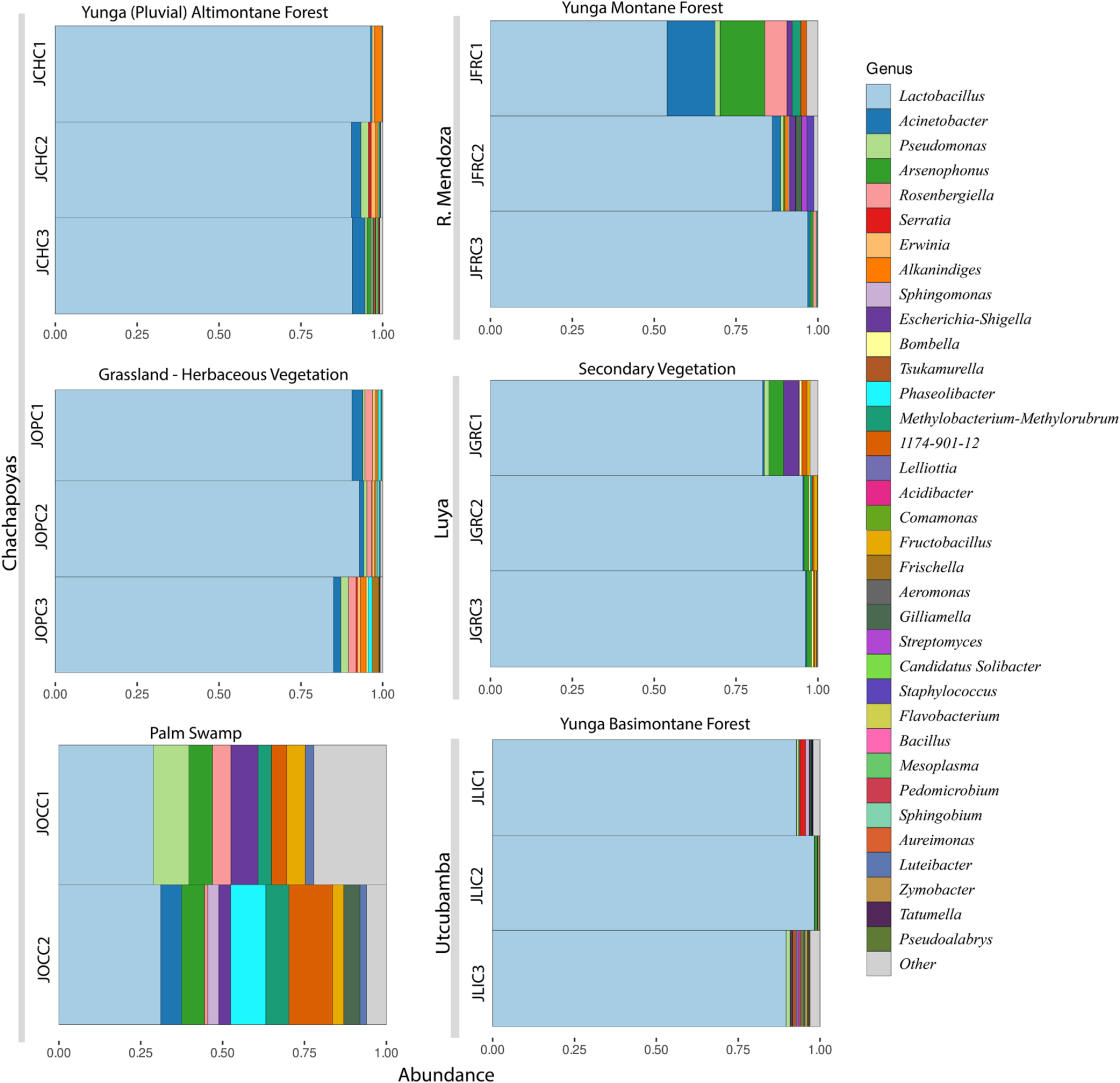
Taxonomic composition at the genus level of the bacterial microbiota associated with honey bee ( *Apis mellifera* ) pollen reserves across six ecosystems in the Amazonas region.

Thirty‐five bacterial genera were identified at the genus level, with *Lactobacillus* (82.38%) being the most abundant, followed by *Acinetobacter* (2.17%) and *Arsenophonus* (2.03%) (Figure 2, Table S7). *Lactobacillus* dominated most ecosystems, particularly in the Y‐bF (93.29%) and Y‐aF (92.85%) (Table S8). Distinct distribution patterns emerged among the other dominant genera: *Acinetobacter* was most prevalent in the Y‐mF (5.28%), while *Arsenophonus* was most abundant in the PS (5.62%). Notably, the PS ecosystem exhibited the greatest variation in genus‐level relative abundance. The least abundant genus, *Tatumella* (0.12%), was detected exclusively in the Y‐bF ecosystem (Figure 2, Table S8).

### 3.3 Taxonomic Assignment and Abundance of Fungal Microbiota Associated With Pollen Reserves

Across the evaluated apiaries, four fungal phyla were identified among the six ecosystems. Basidiomycota dominated the fungal community (99.68%) and were present in all the ecosystems (Figure S5, Table S9). In contrast, the abundance of Ascomycota was minimal (0.099%) and was detected exclusively in the Y‐bF ecosystem (Table S10).

Six fungal taxa were identified at the family level. An unknown family belonging to the Tremellomycetes class (63.87%) was most abundant, followed by Bulleribasidiaceae (34.89%) (Figure S6, Table S11). These two taxa dominated all six ecosystems. In contrast, Saccharomycetaceae was minimally abundant (0.09%) and was present exclusively in the Y‐bF ecosystem (Table S12).

**FIGURE 3:**
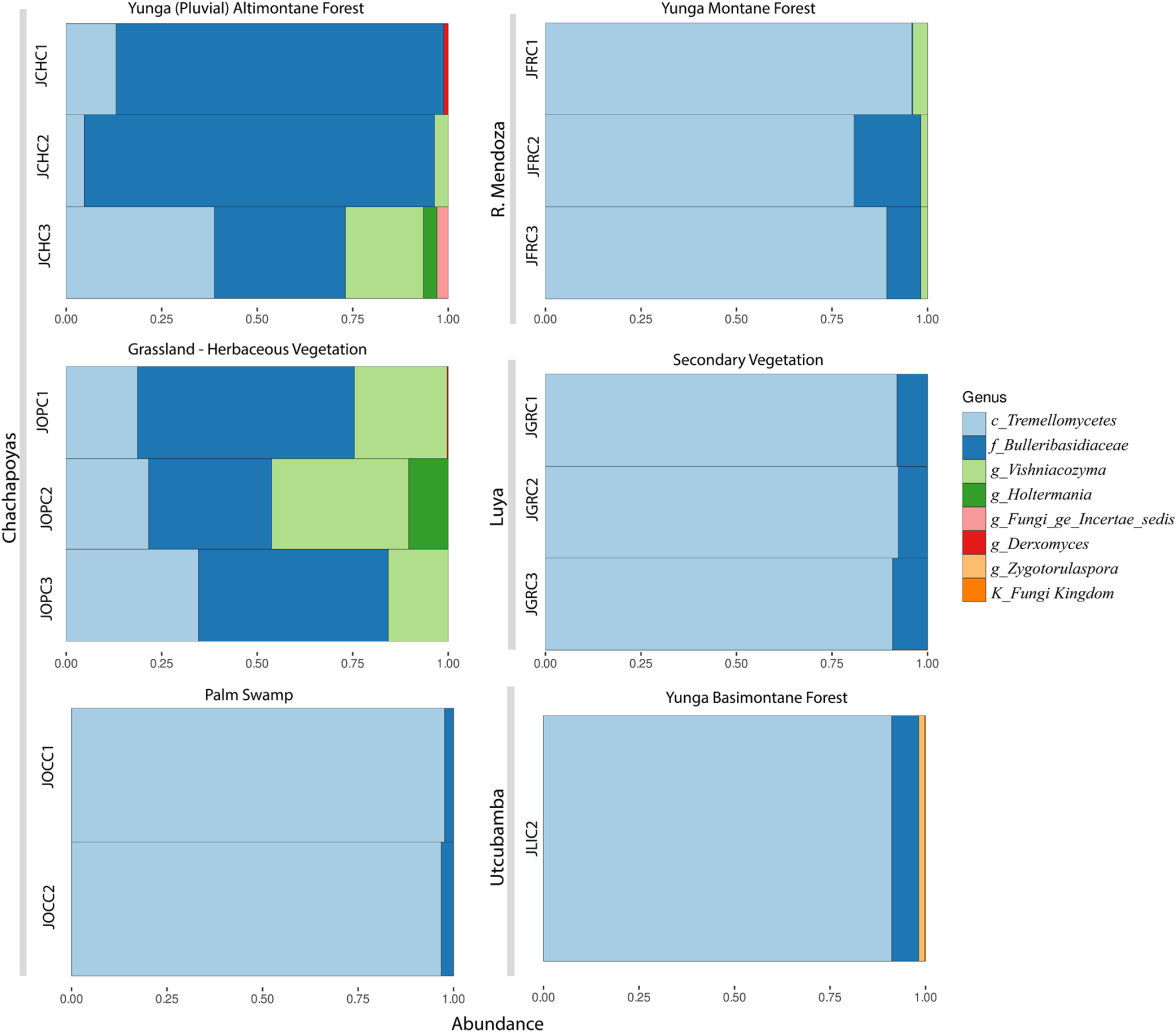
Taxonomic composition at the genus level of the fungal microbiota associated with honey bee ( *Apis mellifera* ) pollen reserves across six ecosystems in the Amazonas region.

Eight taxa were identified at the genus level. An unknown genus from the Tremellomycetes class (63.86%) and an unknown genus from the family Bulleribasidiaceae (27.65%) were most abundant (Figure 3, Table S13). Tremellomycetes‐associated taxa dominated four ecosystems: the PS, Y‐mF, SV areas and Y‐bF. In contrast, an unknown genus from Bulleribasidiaceae was more abundant in two ecosystems: the Y‐aF and GHV areas (Figure 3, Table S14). *Derxomyces* and *Zygotorulaspora* were the least abundant genera (both 0.099%) (Table S14).

### 3.4 Core Microbiota Associated With Pollen Reserves

**FIGURE 4:**
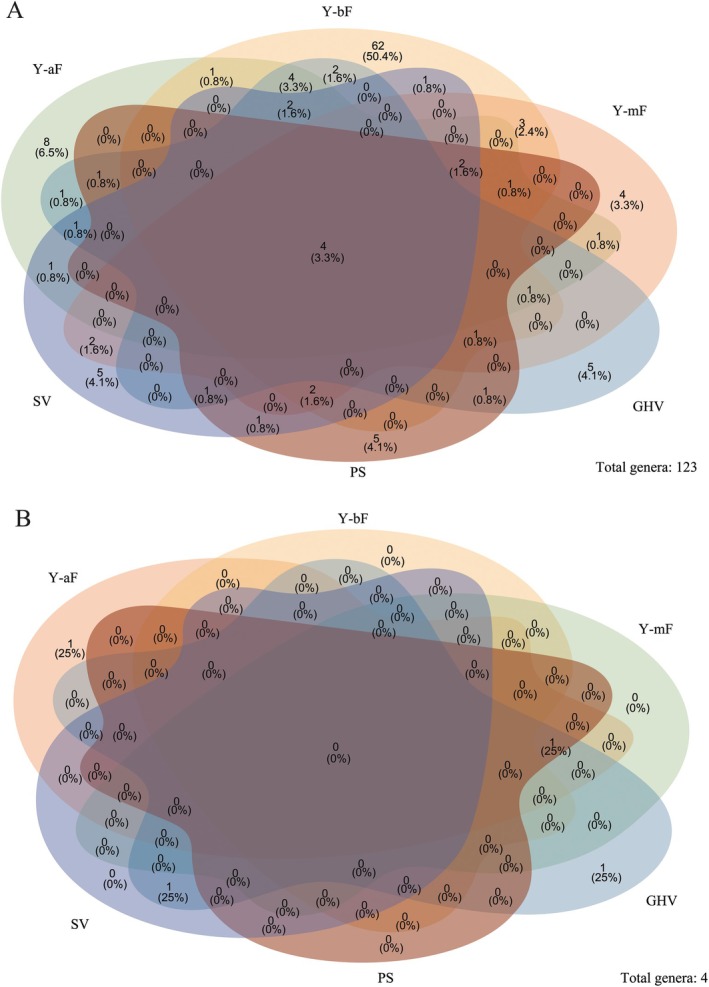
Venn diagram at the genus level showing the core bacterial (A) and fungal microbiota (B) associated with honey bee ( *Apis mellifera* ) pollen reserves across six ecosystems in the Amazonas region. GHV: Grassland‐Herbaceous Vegetation, PS: Palm Swamp, SV: Secondary Vegetation, Y‐aF: Yunga (Pluvial) Altimontane Forest, Y‐bF: Yunga Basimontane Forest, Y‐mF: Yunga Montane Forest.

Among a total of 123 detected bacterial genera, only four (3.3%) were shared across all six ecosystems in the Amazonas region (Figure 4A). The Y‐bF ecosystem contained the greatest number of exclusive genera (62 genera, 50.4%), while the other ecosystems each contained a similar, lower number of exclusive genera, ranging from 4 to 8 (3.3%–6.5%). Genera shared between only two or three ecosystems were scarce (1–3 genera), further indicating limited bacterial community overlap. The bacterial co‐occurrence networks exhibited a highly interconnected structure with multiple well‐defined modules (Figure S7). Interactions within the network were both positively and negatively correlated, suggesting microbial synergism and antagonism. The community of the PS ecosystem exhibited the densest and most highly connected network, with greater interactions, whereas the other ecosystems presented the coexistence of bacterial groups at numerous nodes. *Lactobacillus* predominated (18.2%–66.4%) across the different ecosystems. Other genera, such as *Rosenbergiella*, *Acinetobacter* and *Pseudomonas*, represented varying percentages of nodes in each ecosystem and actively participated in positive associations within specific subnetworks. Genera such as *Erwinia*, *Arsenophonus*, *Tsukamurella* and *Escherichia–Shigella* were represented in relatively low proportions.

For the fungal microbiota, no central set of genera was shared across all six ecosystems of the Amazonas region (Figure 4B). Only the Y‐aF and GHV ecosystems contained one exclusive genus each, while the remaining ecosystems had none. The fungal co‐occurrence network displayed a modular structure comprising three or four defined connected groups (Figure S8). This network was dominated by positive correlations, with no negative interactions observed. The genus *Vishniacozyma* was the most highly represented, accounting for 83.33% of the nodes and occupying central positions within the most interconnected modules.

### 3.5 Diversity Analysis of the Bacterial Microbiota Associated With Pollen Reserves

**FIGURE 5:**
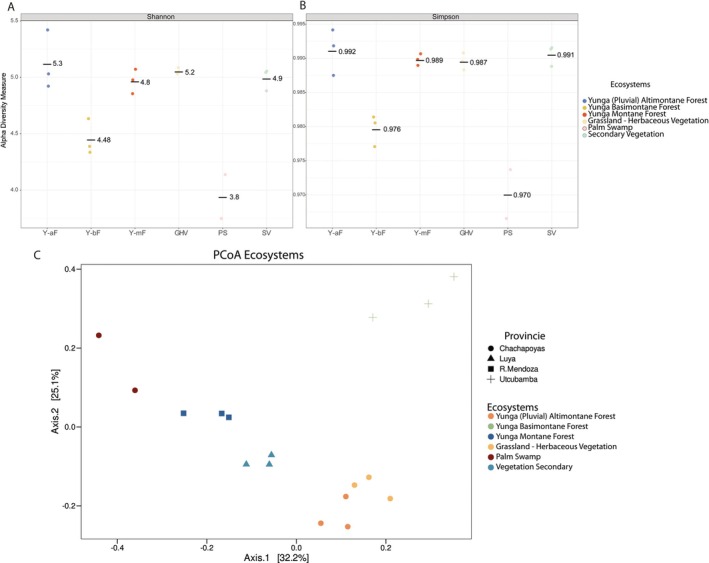
Alpha and beta diversity analyses of bacterial microbiota associated with pollen reserves across six ecosystems in the Amazonas region. Effective numbers of Shannon (A) and Simpson (B) diversity indices and PCoA based on Bray–Curtis dissimilarity (C), explaining 57.3% of the cumulative variance in bacterial community composition.

Effective numbers were used to calculate alpha diversity indices (i.e., the Shannon and Simpson indices) for the bacterial microbiota associated with pollen reserves (Figure 5A,B). Among the six ecosystems, Y‐aF had the greatest diversity (Shannon index = 5.3; Simpson index = 0.993), followed by GHV (Shannon = 5.2; Simpson = 0.988), SV (Shannon = 5.1; Simpson = 0.992) and Y‐mF (Shannon = 4.9; Simpson = 0.990). Y‐bF exhibited moderate diversity (Shannon = 4.3; Simpson = 0.981), whereas PS displayed the lowest diversity values (Shannon = 3.7; Simpson = 0.970) (Figure 5A,B; Table S15).

With respect to the beta diversity analysis of the bacterial microbiota across the six ecosystems, the PCoA based on Bray–Curtis dissimilarity explained 57.3% of the total variability (Axis 1: 32.2%; Axis 2: 25.1%) (Figure 5C). The bacterial communities significantly varied among ecosystems, forming six distinct groups: (i) Y‐aF and GHV; (ii) SV; (iii) Y‐mF; (iv) Y‐bF; and (v‐vi) two separate clusters from PS. Heatmap analysis using GUniFrac distance metrics revealed distinct microbial community patterns among ecosystems (Figure S9). Compared with those from the other ecosystems, the samples from the PS had the greatest dissimilarity (0.5). SV exhibited moderate dissimilarity (0.3), whereas Y‐bF displayed significant dissimilarity (0.5) relative to Y‐mF, SV and PS (Figure S9).

### 3.6 Diversity Analysis of Fungal Microbiota Associated With Pollen Reserves

**FIGURE 6:**
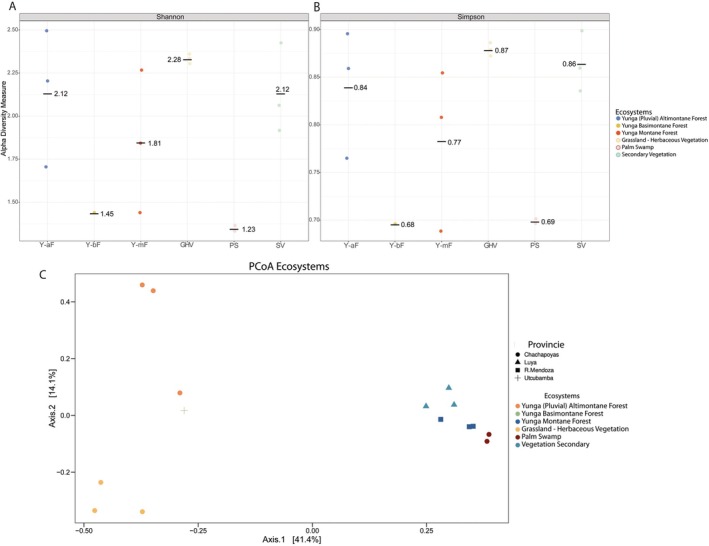
Alpha and beta diversity analyses of fungal microbiota associated with pollen reserves across six ecosystems in the Amazonas region. Shannon (A) and Simpson (B) diversity indices and PCoA based on Bray–Curtis dissimilarity (C), which explained 55.5% of the cumulative variance in fungal community composition.

Effective numbers were used to calculate alpha diversity indices (i.e., Shannon and Simpson) for the fungal microbiota associated with pollen reserves across the six ecosystems (Figure 6A,B). The greatest fungal diversity was detected in GHB (Shannon index = 2.30; Simpson index = 0.885), followed by Y‐aF (Shannon index = 2.28; Simpson index = 0.875). Moderate diversity was observed in Y‐mF (Shannon = 2.10; Simpson = 0.840) and SV (Shannon = 2.15; Simpson = 0.860). In contrast, PS presented the lowest diversity values (Shannon index = 1.30; Simpson index = 0.710) (Figure 6A,B; Table S16).

Beta diversity analysis of the bacterial microbiota across the six ecosystems was assessed using Bray–Curtis dissimilarity and the PCoA explained 55.5% of the total variability (Axis 1: 41.4%; Axis 2: 14.1%) (Figure 6C). Fungal communities clustered into four distinct groups: (i) Y‐mF, SV and PS; (ii) Y‐aF and Y‐bF; (iii) Y‐aF; and (4) PS. Heatmap analysis using UniFrac distance metrics revealed distinct microbial community patterns among ecosystems. Compared with those from the other ecosystems, the samples from Y‐aF and Y‐bF presented the highest dissimilarity values (0.6). The SV samples exhibited moderate dissimilarity (0.2–0.5), whereas the PS samples demonstrated particularly high dissimilarity (0.7) with sample JOPC3 from GHV (Figure S10).

### 3.7 Functional Profile of the Bacterial Microbiota Associated With Pollen Reserves

The bacterial communities associated with pollen reserves exhibited diverse functional profiles, with 189 predicted KOs linked to metabolic pathways. These KOs were functionally annotated and classified into four major categories: cellular processes, environmental information processing, genetic information processing and metabolism (Figure S11). Cellular processes were dominated by functions related to cell growth and death (51%), followed by transport and catabolism (18.8%), cellular motility (17.8%) and prokaryotic cell community interactions (12.5%). The results of the environmental information processing analysis revealed that the majority of the predicted pathways were associated with membrane transport (70%), whereas the remaining 30% were linked to signal transduction. With respect to genetic information processing, the greatest percentage of pathways supported translation (43.6%); followed by replication and repair (31.5%) and folding, sorting and degradation (18.8%) and a small percentage (6.2%) of pathways involved signalling molecules and interactions. Metabolism emerged as the most functionally diverse category, with 12 distinct predicted pathways. Among these pathways, energy metabolism (19.3%) was the most prominent, followed closely by carbohydrate metabolism (18.8%) and amino acid metabolism (18.5%).

**FIGURE 7:**
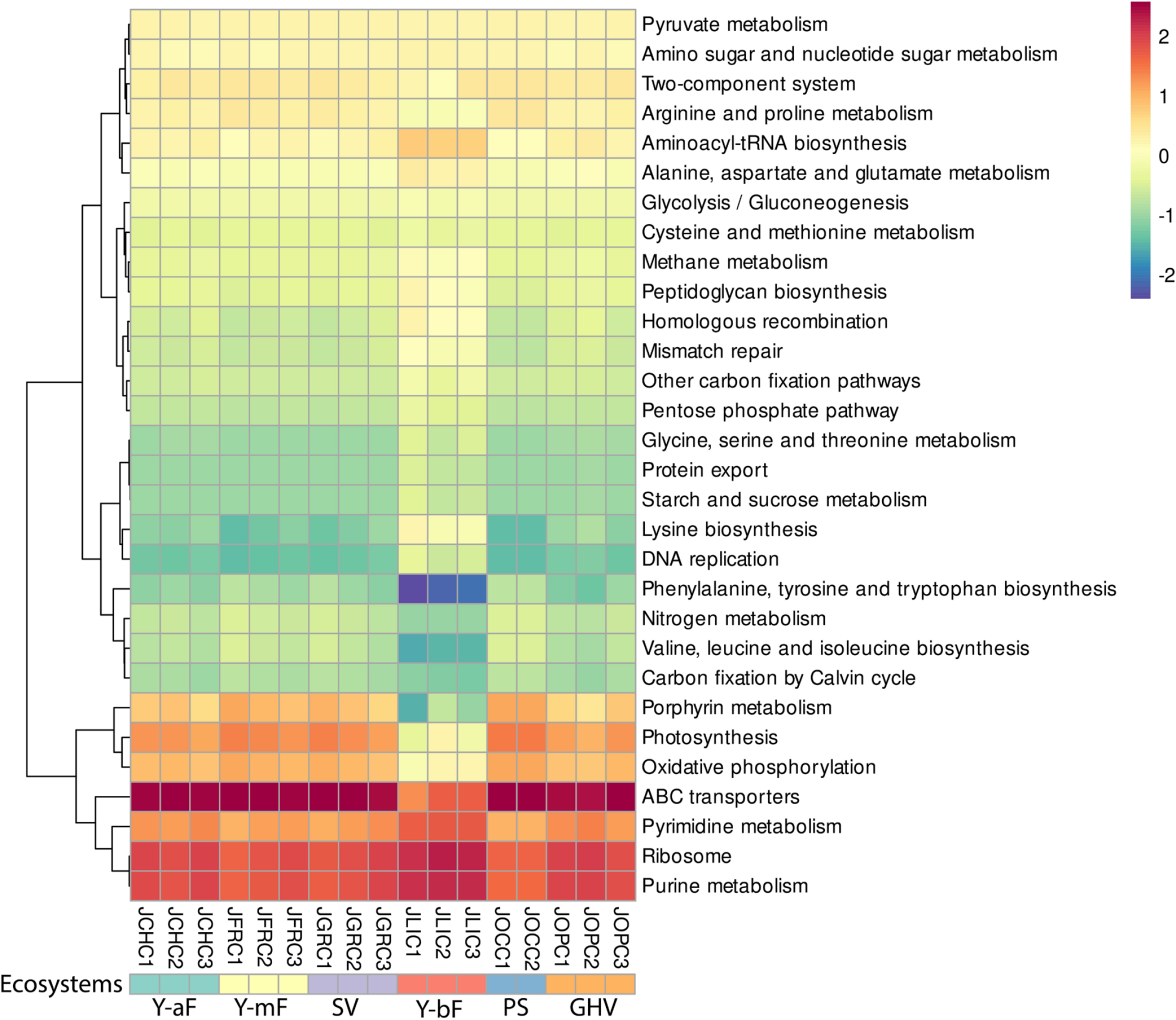
Heatmap displaying predicted metabolic pathways representing the most abundant biological functions and subfunctions in bacterial microbiota associated with bee‐collected pollen reserves across six ecosystems from the Amazonas region. GHV: Grassland‐Herbaceous Vegetation, PS: Palm Swamp, SV: Secondary Vegetation, Y‐aF: Yunga Pluvial: Altimontane Forest, Y‐bF: Yunga Basimontane Forest, Y‐mF: Yunga Montane Forest.

A total of 30 significant metabolic pathways associated with the bacterial microbiota from pollen reserves were identified when the six ecosystems in the Amazonas region were compared (Figure 7). All the ecosystems, except Y‐bF, presented similar expression levels of these 30 metabolic pathways. The pathways with the highest expression levels (represented in red on the heatmap) were primarily ABC transporters, ribosomes and purine metabolism. Those with medium expression levels (orange on the heatmap) included photosynthesis, porphyrin metabolism and oxidative phosphorylation. While most remaining pathways presented low (yellow) to negligible expression levels, aminoacyl‐tRNA biosynthesis was slightly more highly expressed in the Y‐bF ecosystem.

### 3.8 Functional Guild Prediction of the Fungal Microbiota Associated With Pollen Reserves

**FIGURE 8:**
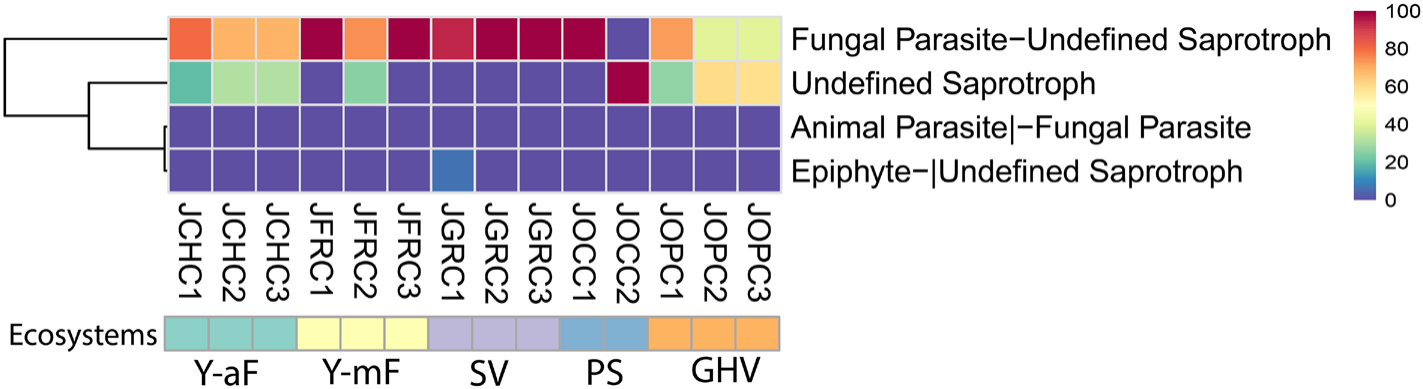
Heatmap displaying the predicted guild functions of the fungal microbiota associated with bee‐collected pollen reserves across six ecosystems from the Amazonas region. GHV: Grassland‐Herbaceous Vegetation, PS: Palm Swamp, SV: Secondary Vegetation, Y‐aF: Yunga Pluvial: Altimontane Forest, Y‐bF: Yunga Basimontane Forest, Y‐mF: and Yunga Montane Forest.

Analysis of fungal functional guilds associated with pollen reserves revealed four predominant trophic groups: (i) fungal parasite–undefined saprotrophs, (ii) undefined saprotrophs, (iii) animal parasites/fungal parasites and (iv) epiphyte–undefined saprotrophs. These functional groups were detected across all the studied ecosystems, although their relative abundances varied significantly (Figure 8). The fungal parasite–undefined saprotroph and undefined saprotroph categories dominated most samples, particularly in the Y‐aF and GHV ecosystems, where they reached peak abundance values. In contrast, the SV and PS ecosystems presented markedly lower representation of these functional groups and maintained consistently low and homogeneous abundance levels. The animal parasite/fungal parasite group maintained a stable but low abundance across all the ecosystems. Notably, the epiphyte–undefined saprotroph group was the least abundant, with near‐zero values in most ecosystems.

## 4 Discussion

Pollen serves not only as a passive pollination vector but also as a dynamic habitat supporting diverse microbial communities, including bacteria and fungi (Cardinale and Schnell 2024). These communities play a pivotal role in flower–pollinator interactions, facilitating the transmission of beneficial microbes (Adler et al. 2021). Specifically, the pollen reserve (beebread) microbiota constitutes essential components for honey bee colony health because it enhances nutritional provisioning, strengthens disease resilience and maintains colony homeostasis (Engel et al. 2012). These microbial communities mediate critical ecological processes, including pollen fermentation, preservation and pathogen suppression (Didaras et al. 2020). However, the taxonomic composition of these communities remains poorly characterized in neotropical ecosystems. A probable limitation of this study is the low microbial biomass typical of stored beebread. This increases the risk of amplifying contaminant DNA, even with stringent negative controls and sterile handling. Despite this, our study provides the first comprehensive analysis of bacterial and fungal communities from pollen reserves across six ecosystems in the Amazonas region using high‐throughput metabarcoding. The results reveal how environmental factors, particularly plant diversity and habitat characteristics, fundamentally shape the composition, diversity and functional potential of the pollen microbiome.

### 4.1 Microbiota Associated With Pollen Reserves

The microbiota composition associated with the pollen reserves was consistent across the five ecosystems for the bacterial communities (i.e., GHV, SV, Y‐aF, YbF and YmF) and among the four ecosystems for the fungal communities (i.e., PS, SV, Y‐bF and Y‐mF) (Figures 2 and 3). This consistency resulted from the predominance of a small number of highly abundant microorganisms, specifically bacteria in the genus *Lactobacillus* and an unclassified fungus belonging to the class Tremellomycetes.

*Lactobacillus* species are gram‐positive, rod‐shaped bacteria that are commonly found in fermented foods, where they contribute to preservation, nutrient availability and flavour development (Batt 2014). Notably, *Lactobacillus* species form biofilms on pollen surfaces, demonstrating their capacity for adhesion and spatial organization within this microhabitat (Ellegaard and Engel 2019). These bacteria play essential roles in honey bee colonies by fermenting pollen components and producing organic acids and bacteriocins, thereby contributing to the preservation of bee bread (Anderson et al. 2023). Furthermore, *Lactobacillus* species exhibit broad‐spectrum antimicrobial activity against both gram‐positive and gram‐negative pathogens through antimicrobial compound production (Georgieva et al. 2015). The widespread distribution of *Lactobacillus* across the six ecosystems in the Amazonas region likely occurs through trophallaxis (mouth‐to‐mouth food exchange), which helps maintain microbial community stability and predominance (De Marco and Farina 2003). For instance, Khiabani et al. (2024) isolated more than 61 strains of lactic acid bacteria from fresh flowers, confirming that flowers serve as reservoirs of *Lactobacillus* that bees acquire during foraging through contact with pollen, nectar and anthers (Engel et al. 2012). Additionally, bee genotype influences the selection of socially transmitted bacterial strains, demonstrating that microbiota composition is not solely determined by external environmental factors (Liberti et al. 2022). Consequently, honey bee microbial communities show relatively stable profiles across distinct environments, although certain shifts or declines may occur, possibly associated with human activities such as pesticide use and habitat fragmentation (Anderson et al. 2023). This bacterial homogeneity was confirmed across the evaluated ecosystems, with the exception of the PS. The lower prevalence of *Lactobacillus* in the PS may be related to the area's characteristic floristic composition. Unlike the other five ecosystems studied, the PS is characterized by homogeneous vegetation dominated by a few palm species (MINAM 2018). This results in a reduced diversity of floral resources, thereby limiting opportunities for the acquisition and transmission of *Lactobacillus* from different plant species (Manirajan et al. 2018). Like what has been observed in agricultural landscapes dominated by monocultures, this lower degree of floral heterogeneity may decrease the stability of microbial communities and reduce the abundance of beneficial taxa (Parreño et al. 2024).

On the other hand, the unclassified fungal genus from the class Tremellomycetes comprises basidiomycete fungi that are widely distributed in terrestrial environments and exhibit ecological diversity, including saprotrophic, mycoparasitic, lichenicolous and even animal pathogen species (Millanes et al. 2011). These fungi have been found in the guts of phytophagous insects, where they may contribute to gut microbiome modulation and lignocellulosic compound degradation (Doğan et al. 2024). Although information on this fungal class remains scarce and its ecological function is not fully understood, its association with *Apis mellifera* could result from exposure to flowers, nectar and pollen contaminated with Tremellomycetes, either naturally or as a result of storage in hives. The lower abundance of Tremellomycetes in the Y‐aF is likely associated with the distinct environmental and floral characteristics of these habitats. The Y‐aF, located at higher elevations (2500–3800 m a.s.l.), is characterized by low temperatures, high humidity and frequent cloud cover. This microclimate may be less favourable for Tremellomycetes proliferation (Millanes et al. 2011). Therefore, the distribution pattern of Tremellomycetes observed in this study appears to be strongly influenced by floral composition and habitat complexity.

The limited core bacterial and fungal microbiota shared across the six ecosystems reflects a high degree of microbial heterogeneity. This pattern indicates that pollen reserve microbiota communities are primarily structured by local environmental conditions and respond to variations in vegetation, climate and the broader ecological context (Vannette 2026). Furthermore, pollen reserve itself functions as a dynamic microhabitat where the bacterial and fungal microbiota are assembled from multiple environmental sources, including floral surfaces, air, soil and pollinators. In this system, *Apis mellifera* acts not only as a dispersal vector but also as a selective filter, modulating which microorganisms persist during foraging (McFrederick et al. 2017). Despite the taxonomic heterogeneity, the bacterial co‐occurrence network exhibited a highly organized and nonrandom structure, which is consistent with patterns reported for other microbiomes associated with floral resources and bee‐stored foods (Manirajan et al. 2018). In these systems, taxonomically variable communities converge toward stable networks governed by key taxa (e.g., *Lactobacillus*) (Dew et al. 2020), shaped by strong functional filters. In this scenario, only microorganisms capable of tolerating acidic conditions, intense competition and nutrient limitation can persist and integrate into the community network (Anderson et al. 2023; Disayathanoowat et al. 2020).

### 4.2 Diversity Analysis of the Microbiota Associated With Pollen Reserves

Our analysis revealed that ecosystem type is a fundamental driver shaping the structure of both bacterial and fungal communities associated with pollen reserves in the Amazonas region. However, the two microbial kingdoms respond in distinct ways, highlighting their differing ecological sensitivities and assembly processes (Rao et al. 2021). The most striking and consistent finding across both the bacterial and fungal analyses was the significantly reduced alpha diversity in the PS ecosystem. This pattern aligns with its uniform vegetation and water‐saturated, hydromorphic soils (MINAM 2018), which act as strong environmental filters, limiting the range of microbial taxa that can successfully colonize pollen (Disayathanoowat et al. 2020). This finding supports the broader ecological principle that simplified habitats, such as monocultures or ecosystems with low plant diversity, often sustain lower microbial richness than more complex environments do (Muñoz‐Colmenero et al. 2020). Beyond this shared pattern, the responses of bacteria and fungi diverged. With respect to bacteria, the greatest diversity was detected in the Y‐aF, suggesting that the complex, multistrata vegetation of this mature forest provides an exceptionally heterogeneous niche space for a wide variety of bacterial taxa (Keller et al. 2021). In contrast, fungal diversity peaked in the GHV ecosystem. This difference may reflect fundamental differences in lifestyle. With its dense root networks and potentially high turnover of organic matter (MINAM 2018), the GHV ecosystem might offer a more favourable substrate for diverse saprotrophic or plant‐pathogenic fungi, which subsequently colonize pollen.

Additionally, our findings demonstrate that the stored pollen serves as a microhabitat whose microbial inhabitants are powerfully filtered by the ecosystem. This kingdom‐specific structuring is further corroborated by the beta diversity results. The strong separation of bacterial communities in the PCoA and the distinct clustering in the heatmap confirm that each ecosystem fosters a unique bacterial assemblage. The fungal communities, while also significantly structured by the ecosystem, displayed different alliance patterns. The clustering of the Y‐mF, SV and PS samples suggests that fungal assembly is influenced by a different set of environmental or host factors. Furthermore, the high dissimilarity observed for the Y‐aF and Y‐bF samples in the fungal heatmap points to a high degree of heterogeneity or endemicity within these forests for fungi (Geml et al. 2014). This is a pattern not observed in the bacterial data. This could be linked to the strong host‐specific relationships many fungi form with plants (Schön et al. 2023), which would be directly reflected in the pollen reserve microbiome. While both bacterial and fungal communities are shaped by these broad environmental gradients, they exhibit distinct diversity peaks and community structures. These findings confirm that bacteria and fungi respond to different aspects of the ecosystem, such as vegetation complexity, soil properties and host plant identity (Panico et al. 2025). Additionally, the consistently low diversity in the PS highlights its vulnerability, potentially exacerbated by anthropogenic disturbances such as selective logging, which can homogenize vegetation and further deplete microbial richness (Nguyen and Rehan 2023).

### 4.3 Functional Profiles of the Bacterial and Fungal Communities Associated With Pollen Reserves

This study confirmed that the microbial communities inhabiting pollen reserves have conserved functions, despite taxonomic variations across ecosystems. This functional conservation appears to be a defining feature of the pollen reserve microbiome, shaped by the stringent selective pressures of the hive environment, which demands stability for food preservation and colony health. The bacterial functional profile is predominantly geared toward survival, proliferation and resource exploitation within this unique niche. The high expression of ABC transporters, ribosome biogenesis and purine metabolism confirms the presence of a metabolically active community. These pathways are essential for nutrient import from the pollen substrate, rapid protein synthesis and energy generation, allowing bacteria to capitalize on the nutrient richness of the pollen (Khan et al. 2020). The significant representation of energy and carbohydrate metabolism further underscores their role as primary consumers in this microecosystem, directly catabolizing pollen‐derived sugars and lipids. While this core metabolic signature is consistent across most ecosystems, the distinct upregulation of aminoacyl‐tRNA biosynthesis in Y‐bF suggests a unique physiological state, potentially indicating higher protein turnover rates or a response to specific environmental stresses not encountered elsewhere (Maccaro et al. 2024).

Conversely, fungal guilds outline a clear trophic structure centred on decomposition and recycling. The dominance of saprotrophic guilds (i.e., fungal parasite–undefined saprotrophs and undefined saprotrophs) across all ecosystems highlights their critical role in breaking down complex organic matter in pollen (Zimny et al. 2024). However, the significant fluctuation in their abundance (peaking in the Y‐aF and GHV ecosystems but remaining low in the SV and PS ecosystems) indicates that local environmental factors or pollen type can modulate the intensity of saprotrophic activity. The stable, low abundance of the animal parasite/fungal parasite guild suggests a consistent, minor niche for mycoparasitism or predation on other pollen‐dwelling microfauna (O'Keeffe et al. 2021). Notably, the near‐absence of epiphytic fungi indicates that the pollen reserve microbiome is distinct from the phyllosphere (Yao et al. 2019), representing a protected, internal environment rather than a surface habitat.

The synergy between these bacterial and fungal functions is crucial for hive health (Disayathanoowat et al. 2020). Bacteria likely drive the initial mobilization of simple nutrients, whereas fungi may target more recalcitrant compounds. This division of labor, maintained through functional redundancy (Vigil et al. 2024; Carrion et al. 2025), ensures ecosystem resilience. The consistent presence of these core processes, regardless of ecosystem origin and microbiota diversity, demonstrates that the hive imposes a powerful filter, selecting for a conserved metabolic profile that guarantees pollen preservation and microbial equilibrium (Dew et al. 2020). Accordingly, the pollen reserves are a functionally stable microecosystem where conserved microbial processes performed by taxonomically flexible communities support the greater metabolic needs of the bee hive.

## 5 Conclusion

On the basis of a comprehensive, high‐throughput metabarcoding analysis across six ecosystems in the Amazonas region, this study revealed that pollen reserve functions as a dynamic microhabitat hosting taxonomically diverse yet functionally conserved bacterial and fungal communities. The assembly of this pollen reserve microbiota is fundamentally shaped by local environmental filters, including ecosystem type, vegetation complexity and floral resources. This resulted in high beta diversity and a limited number of shared microbes. Despite this taxonomic heterogeneity, a robust functional core is maintained, characterized by bacterial metabolic pathways for nutrient utilization and fungal guilds dedicated to decomposition. This functional redundancy ensures ecosystem resilience and supports key hive processes such as pollen preservation and nutrient cycling. The pronounced reduction in microbial diversity within the simplified PS ecosystem underscores the vulnerability of these communities to habitat homogenization. These findings provide the first integrated portrait of the pollen reserve microbiome in a neotropical biodiversity hotspot, highlighting its intricate formation through environmental selection, pollinator‐mediated filtering and functional adaptation to the hive environment.

## Author Contributions

**Jessica C. Llaja:** investigation, writing – original draft, methodology, formal analysis, data curation, software, writing – review and editing. **Fernando Chuquizuta:** investigation, visualization, formal analysis, software, data curation, writing – review and editing. **Martha S. Calderon:** conceptualization, investigation, writing – original draft, writing – review and editing, validation, supervision, data curation, formal analysis. **Jois V. Carrion:** investigation, formal analysis, software, data curation, writing – review and editing. **Danilo E. Bustamante:** conceptualization, investigation, funding acquisition, writing – original draft, writing – review and editing, visualization, validation, supervision, data curation, formal analysis. **Johann E. Oyola:** investigation, methodology, formal analysis, validation, writing – review and editing.

## Funding

This research was funded by the Peruvian National Council for Science, Technology, and Technological Innovation (CONCYTEC) under the projects APIGEN (N° PE501083491‐2023‐PROCIENCIA) and MiCroResi (N° PE501079652‐2022‐PROCIENCIA). This study was also funded by Universidad Nacional Toribio Rodríguez de Mendoza under the project CEINFOR (CUI N° 2315092). This study was also funded by Vicerrectorado de Investigacion (VRIN) of the Universidad Nacional Toribio Rodríguez de Mendoza.

## Conflicts of Interest

The authors declare no conflicts of interest.

## Supporting information


**Figure S1:** The histograms display the quality values (quality score and Phred score) of the reads for bacterial communities from pollen reserves. Quality control histograms were generated for the V3–V4 region of the 16S rRNA gene (300 bp) (a), forward reads (b) and reverse reads (c). After quality filtering, all the sequences had a final length of 270 bp.
**Figure S2:** The histograms display the quality values (quality score and Phred score) of the reads for fungal communities from pollen reserves. Quality control histograms were generated for the ITS2 region (300 bp) (a), forward reads (b) and reverse reads (c). After quality filtering, all the sequences had a final length of 275 bp.
**Figure S3:** Taxonomic composition at the phylum level of the bacterial microbiota associated with honey bee ( *Apis mellifera* ) pollen reserves across six ecosystems in the Amazonas region.
**Figure S4:** Taxonomic composition at the family level of the bacterial microbiota associated with honey bee ( *Apis mellifera* ) pollen reserves across six ecosystems in the Amazonas region.
**Figure S5:** Taxonomic composition at the phylum level of the fungal microbiota associated with honey bee ( *Apis mellifera* ) pollen reserves across six ecosystems in the Amazonas region.
**Figure S6:** Taxonomic composition at the family level of the fungal microbiota associated with honey bee ( *Apis mellifera* ) pollen reserves across six ecosystems in the Amazonas region.
**Figure S7:** Genus‐level co‐occurrence network of the bacterial microbiota associated with honey bee ( *Apis mellifera* ) pollen reserves across six ecosystems in the Amazonas region.
**Figure S8:** Genus‐level co‐occurrence network of the fungal microbiota associated with honey bee ( *Apis mellifera* ) pollen reserves across six ecosystems in the Amazonas region.
**Figure S9:** Heatmap analysis of bacterial microbiota associated with pollen reserves across six ecosystems, based on GUniFrac pairwise distances. Ecosystems include the Yunga (Pluvial) Altimontane Forest (Y‐aF), the Yunga Montane Forest (Y‐mF), secondary vegetation (SV) areas, the Yunga Basimontane Forest (Y‐bF), the Palm Swamp (PS) and grassland‐herbaceous vegetation (GHV) areas.
**Figure S10:** Heatmap analysis of fungal microbiota associated with pollen reserves across six ecosystems, based on GUniFrac pairwise distances. Ecosystems include the Yunga (Pluvial) Altimontane Forest (Y‐aF), the Yunga Montane Forest (Y‐mF), secondary vegetation (SV) areas, the Yunga Basimontane Forest (Y‐bF), the Palm Swamp (PS) and grassland‐herbaceous vegetation (GHV) areas.
**Figure S11:** Pathways related to the biological functions and subfunctions of the bacterial microbiota associated with bee pollen reserves across six ecosystems in the Amazonas region.

**Table S1:** Coordinates and locations of the apiaries evaluated in this study.
**Table S2:** Read quality processing results for the microbiota associated with pollen reserves.
**Table S3:** Total taxonomic composition at the phylum level of bacteria associated with pollen reserves in the Amazonas region.
**Table S4:** Taxonomic composition at the phylum level of bacteria associated with pollen reserves in the six ecosystems in the Amazonas region.
**Table S5:** Total taxonomic composition at the family level of bacteria associated with pollen reserves in the Amazonas region.
**Table S6:** Taxonomic composition at the family level of bacteria associated with pollen reserves in the six ecosystems in the Amazonas region.
**Table S7:** Total taxonomic composition at the genus level of bacteria associated with pollen reserves in the Amazonas region.
**Table S8:** Taxonomic composition at the genus level of bacteria associated with pollen reserves in the six ecosystems in the Amazonas region.
**Table S9:** Total taxonomic composition at the phylum level of fungi associated with pollen reserves in the Amazonas region.
**Table S10:** Taxonomic composition at the phylum level of fungi associated with pollen reserves in the six ecosystems in the Amazonas region.
**Table S11:** Total taxonomic composition at the family level of fungi associated with pollen reserves in the Amazonas region.
**Table S12:** Taxonomic composition at the family level of fungi associated with pollen reserves in the six ecosystems in the Amazonas region.
**Table S13:** Total taxonomic composition at the genus level of fungi associated with pollen reserves in the Amazonas region.
**Table S14:** Taxonomic composition at the genus level of fungi associated with pollen reserves in the six ecosystems in the Amazonas region.
**Table S15:** Alpha diversity indices of bacteria associated with pollen reserves across six ecosystems in the Amazonas region.
**Table S16:** Alpha diversity indices of fungi associated with pollen reserves across six ecosystems in the Amazonas region.

## Data Availability

The data that support the findings of this study are openly available in National Center for Biotechnology Information (NCBI) at https://www.ncbi.nlm.nih.gov/genbank/, reference number BioProject numbers PRJNA1415230 and PRJNA1415238.
