# Supplementary material for: Hidden Microbiota Inhabiting in Pollen Reserves of Honey Bee (Apis mellifera) From Amazonas Region Revealed by DNA Metabarcoding
Source: Environ Microbiol Rep. 2026 Jul 22;18(4):e70392. doi: 10.1111/1758-2229.70392 (PMC13392498; doi:10.1111/1758-2229.70392)
Supplement: Supplementary file 1 — Figure S1: The histograms display the quality values (quality score and Phred score) of the reads for bacterial communities from pollen reserves. Quality control histograms were generated for the V3–V4 region of the 16S rRNA gene (300 bp) (a), forward reads (b) and reverse reads (c). After quality filtering, all the sequences had a final length of 270 bp. Figure S2: The histograms display the quality values (quality score and Phred score) of the reads for fungal communities from pollen reserves. Quality control histograms were generated for the ITS2 region (300 bp) (a), forward reads (b) and reverse reads (c). After quality filtering, all the sequences had a final length of 275 bp. Figure S3: Taxonomic composition at the phylum level of the bacterial microbiota associated with honey bee ( Apis mellifera ) pollen reserves across six ecosystems in the Amazonas region. Figure S4: Taxonomic composition at the family level of the bacterial microbiota associated with honey bee ( Apis mellifera ) pollen reserves across six ecosystems in the Amazonas region. Figure S5: Taxonomic composition at the phylum level of the fungal microbiota associated with honey bee ( Apis mellifera ) pollen reserves across six ecosystems in the Amazonas region. Figure S6: Taxonomic composition at the family level of the fungal microbiota associated with honey bee ( Apis mellifera ) pollen reserves across six ecosystems in the Amazonas region. Figure S7: Genus‐level co‐occurrence network of the bacterial microbiota associated with honey bee ( Apis mellifera ) pollen reserves across six ecosystems in the Amazonas region. Figure S8: Genus‐level co‐occurrence network of the fungal microbiota associated with honey bee ( Apis mellifera ) pollen reserves across six ecosystems in the Amazonas region. Figure S9: Heatmap analysis of bacterial microbiota associated with pollen reserves across six ecosystems, based on GUniFrac pairwise distances. Ecosystems include the Yunga (Pluvial) Altimontane Forest (Y‐ [file EMI4-18-e70392-s002.docx]

**Supplemental Figures**


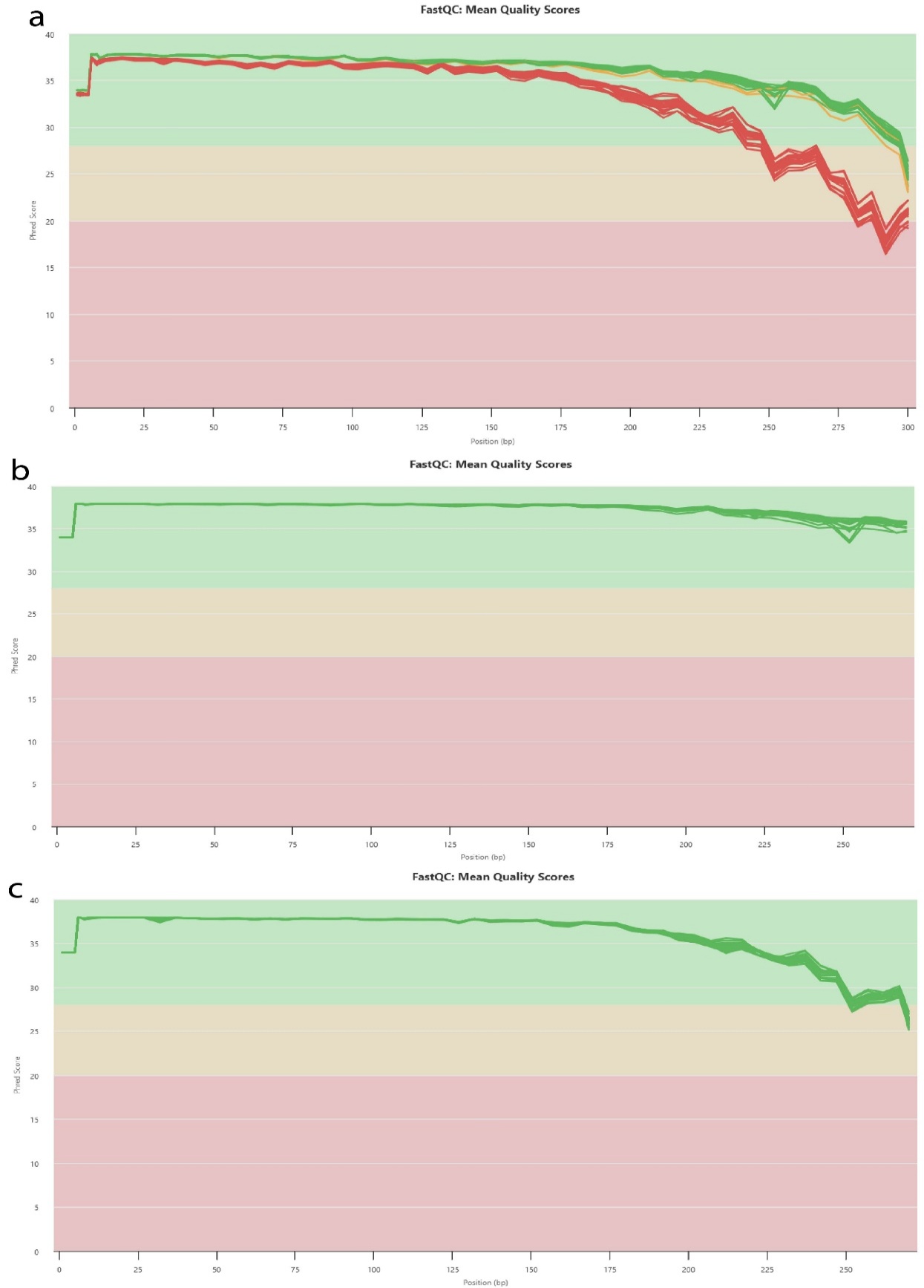


Figure S1. The histograms display the quality values (quality score and Phred score) of the reads for bacterial communities from pollen reserves. Quality control histograms were generated for the V3-V4 region of the 16S rRNA gene (300 bp) (a), forward reads (b), and reverse reads (c). After quality filtering, all the sequences had a final length of 270 bp.

**
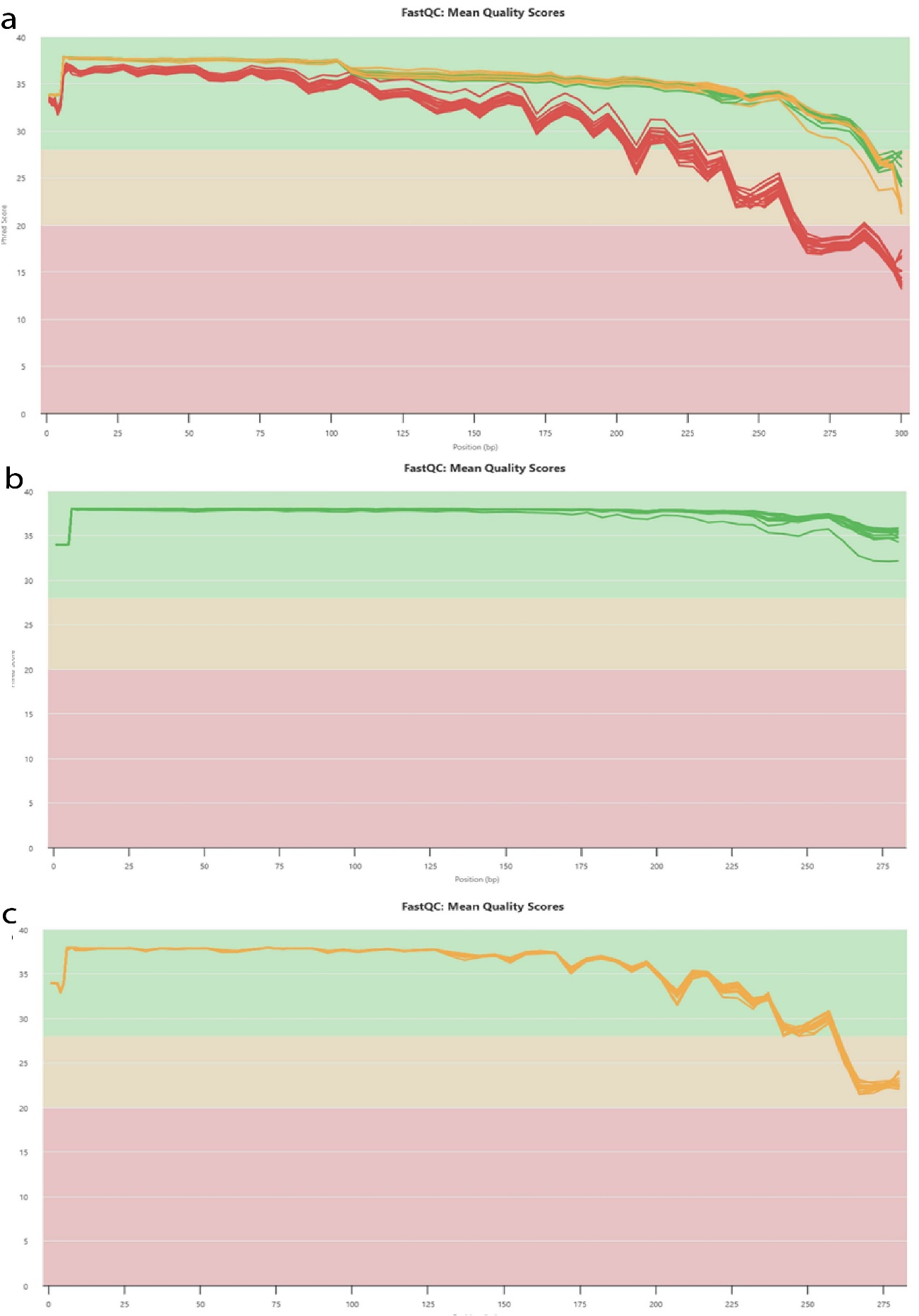
**

**Figure S2.** The histograms display the quality values (quality score and Phred score) of the reads for fungal communities from pollen reserves. Quality control histograms were generated for the ITS2 region (300 bp) (a), forward reads (b), and reverse reads (c). After quality filtering, all the sequences had a final length of 275 bp.


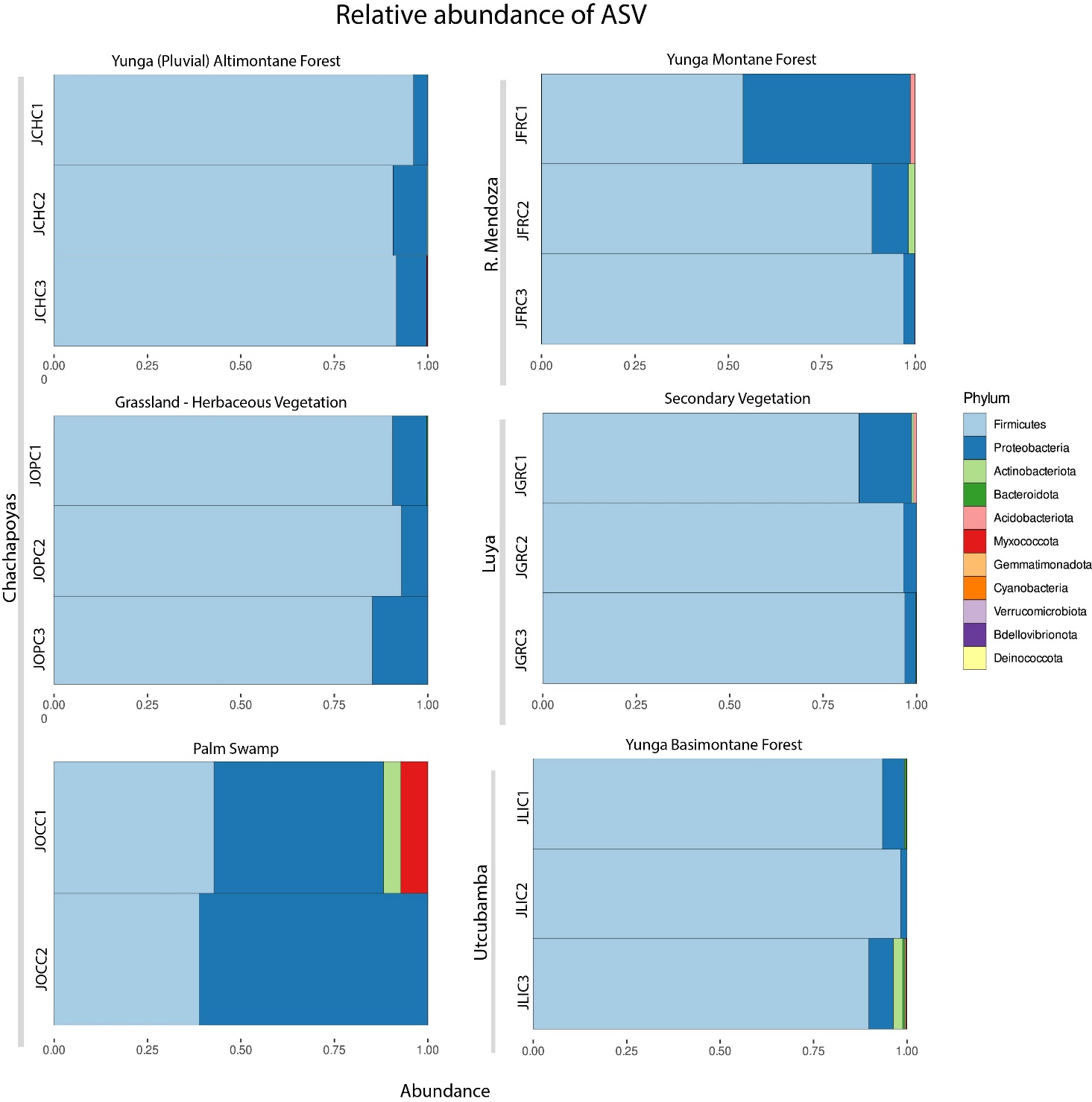


**Figure S3.** Taxonomic composition at the phylum level of the bacterial microbiota associated with honey bee (*Apis mellifera*) pollen reserves across six ecosystems in the Amazonas region.


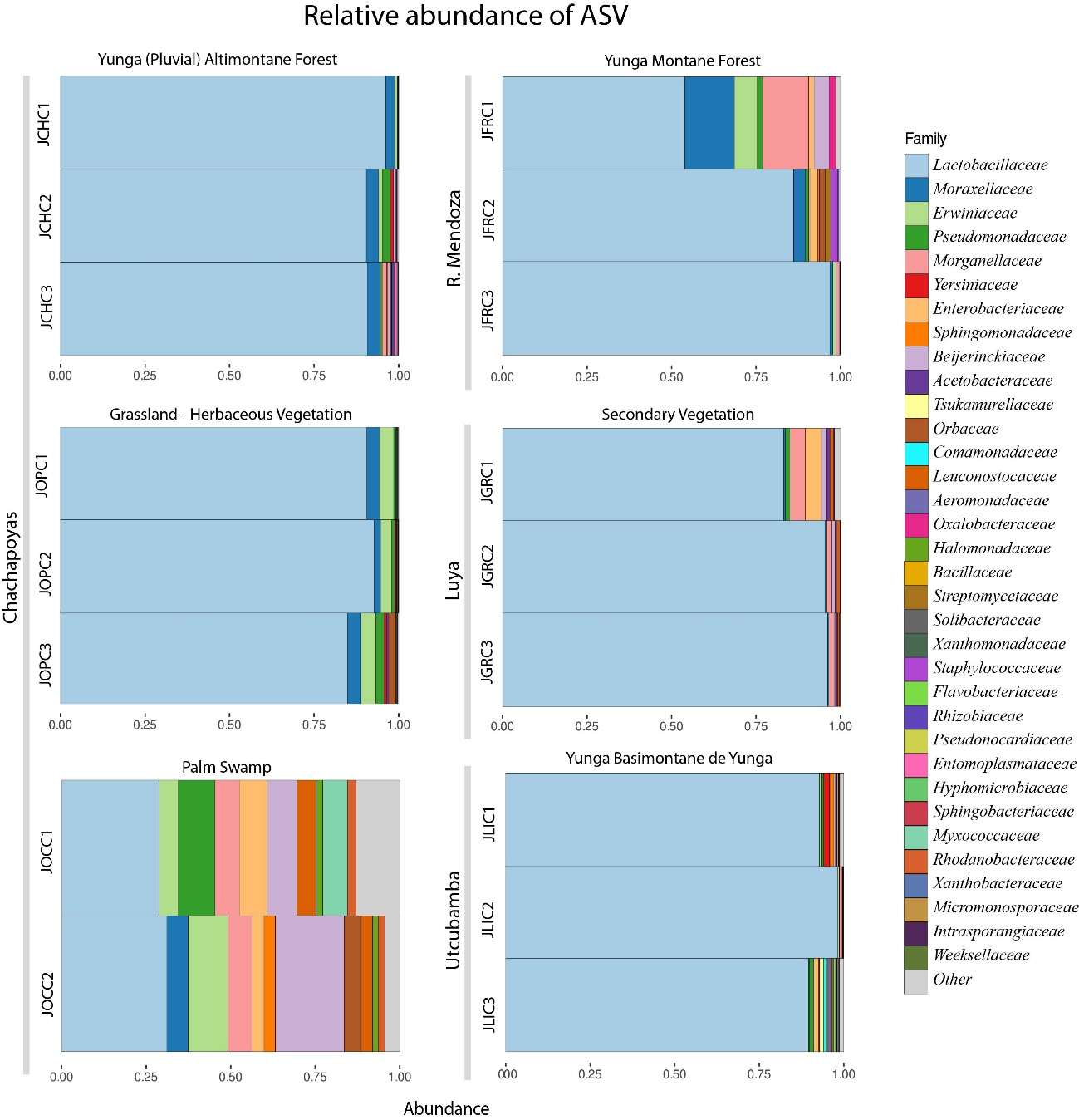


**Figure S4.** Taxonomic composition at the family level of the bacterial microbiota associated with honey bee (*Apis mellifera*) pollen reserves across six ecosystems in the Amazonas region.


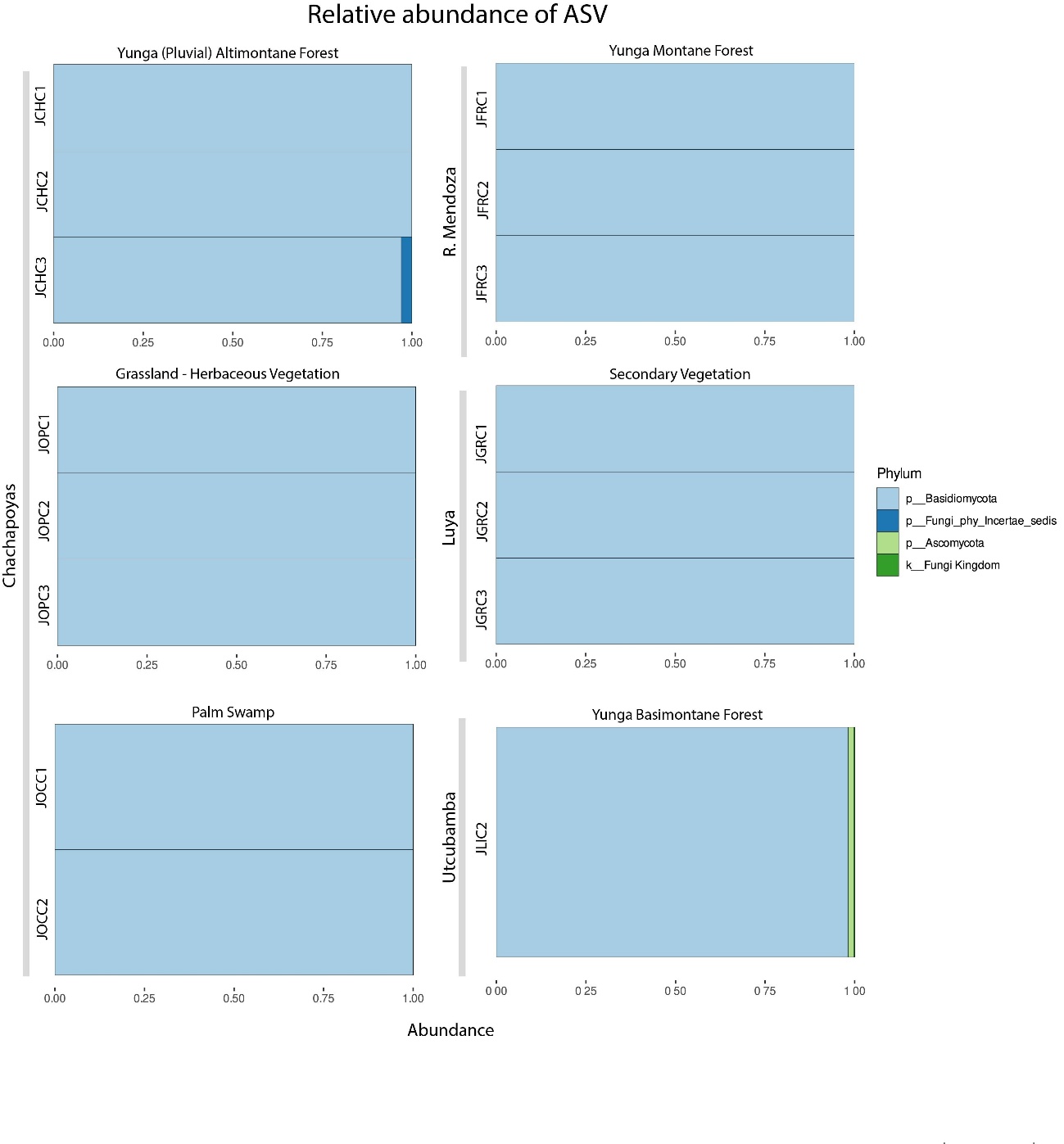


**Figure S5.** Taxonomic composition at the phyllum level of the fungal microbiota associated with honey bee (*Apis mellifera*) pollen reserves across six ecosystems in the Amazonas region.


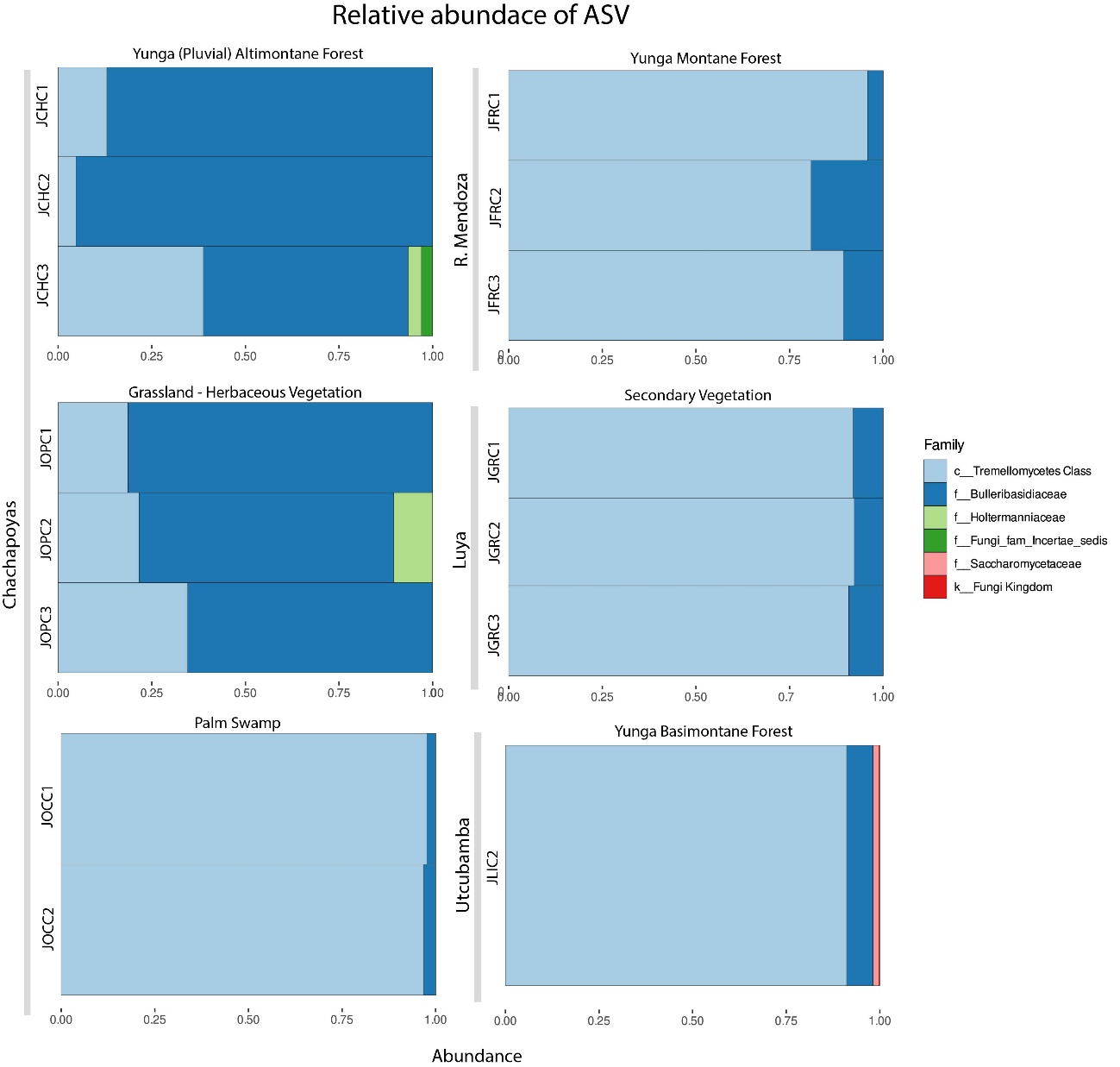


**Figure S6.** Taxonomic composition at the family level of the fungal microbiota associated with honey bee (*Apis mellifera*) pollen reserves across six ecosystems in the Amazonas region.


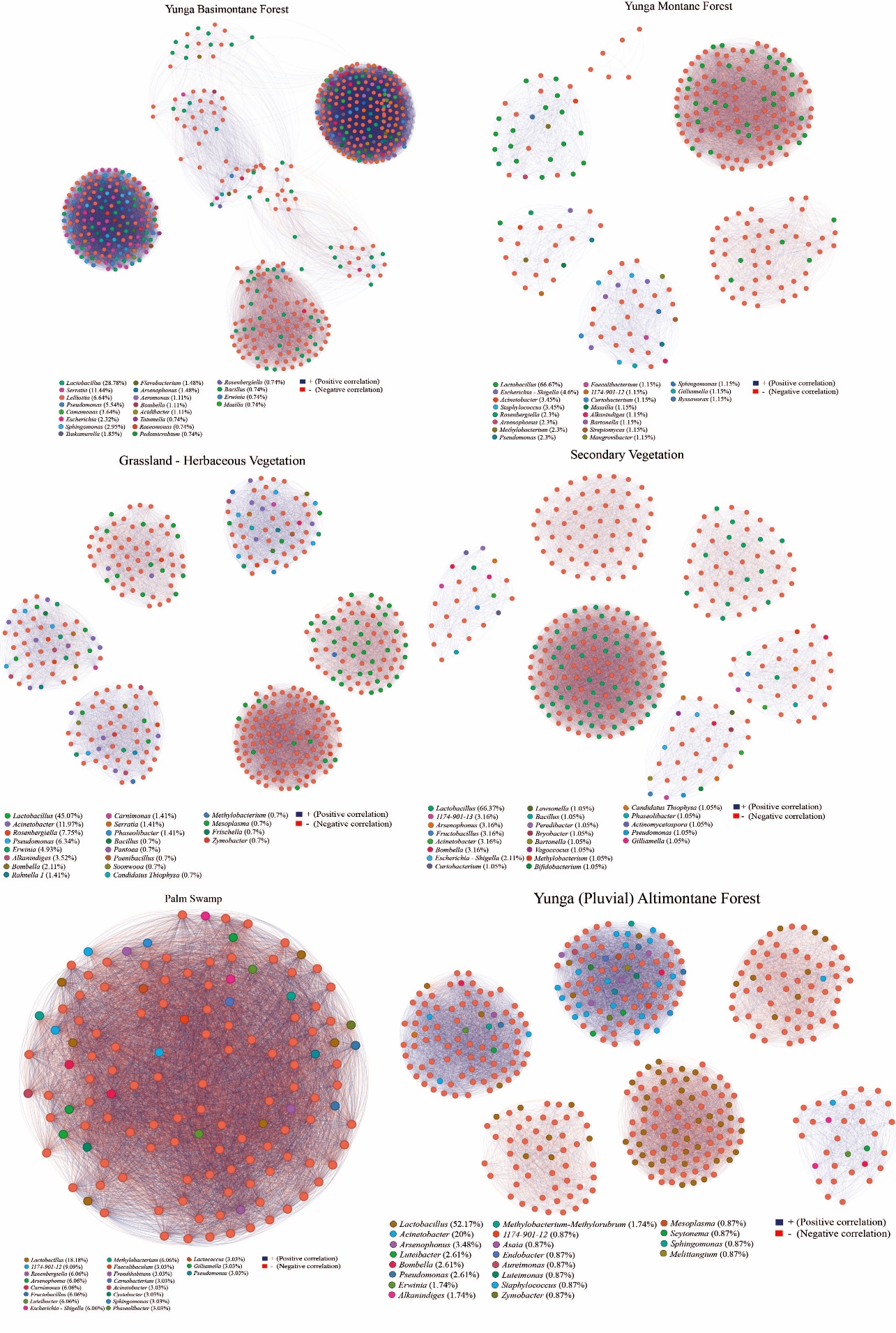


**Figure S7.** Genus-level co-occurrence network of the bacterial microbiota associated with honey bee (*Apis mellifera*) pollen reserves across six ecosystems in the Amazonas region.

**
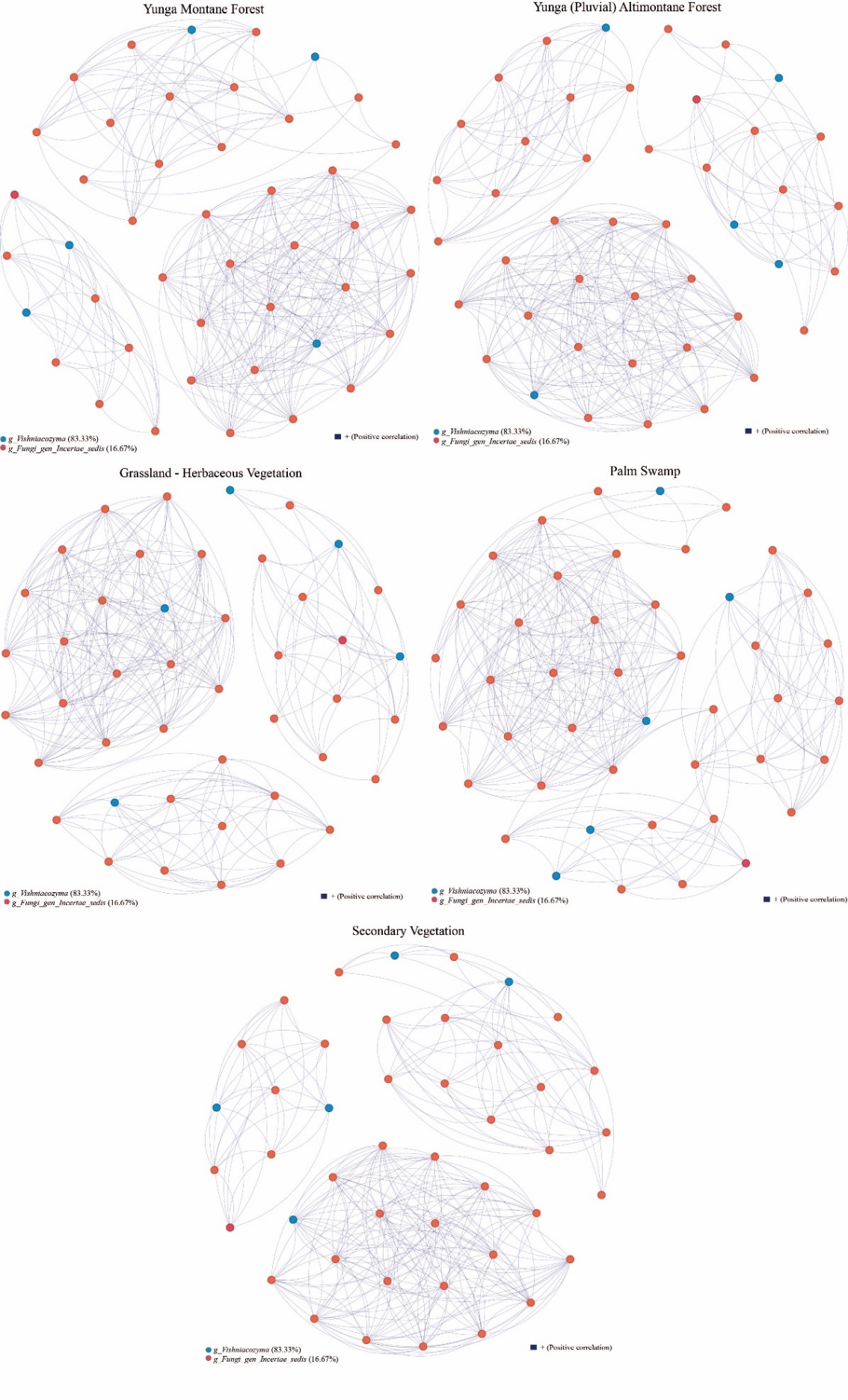
**

**Figure S8.** Genus-level co-occurrence network of the fungal microbiota associated with honey bee (*Apis mellifera*) pollen reserves across six ecosystems in the Amazonas region.


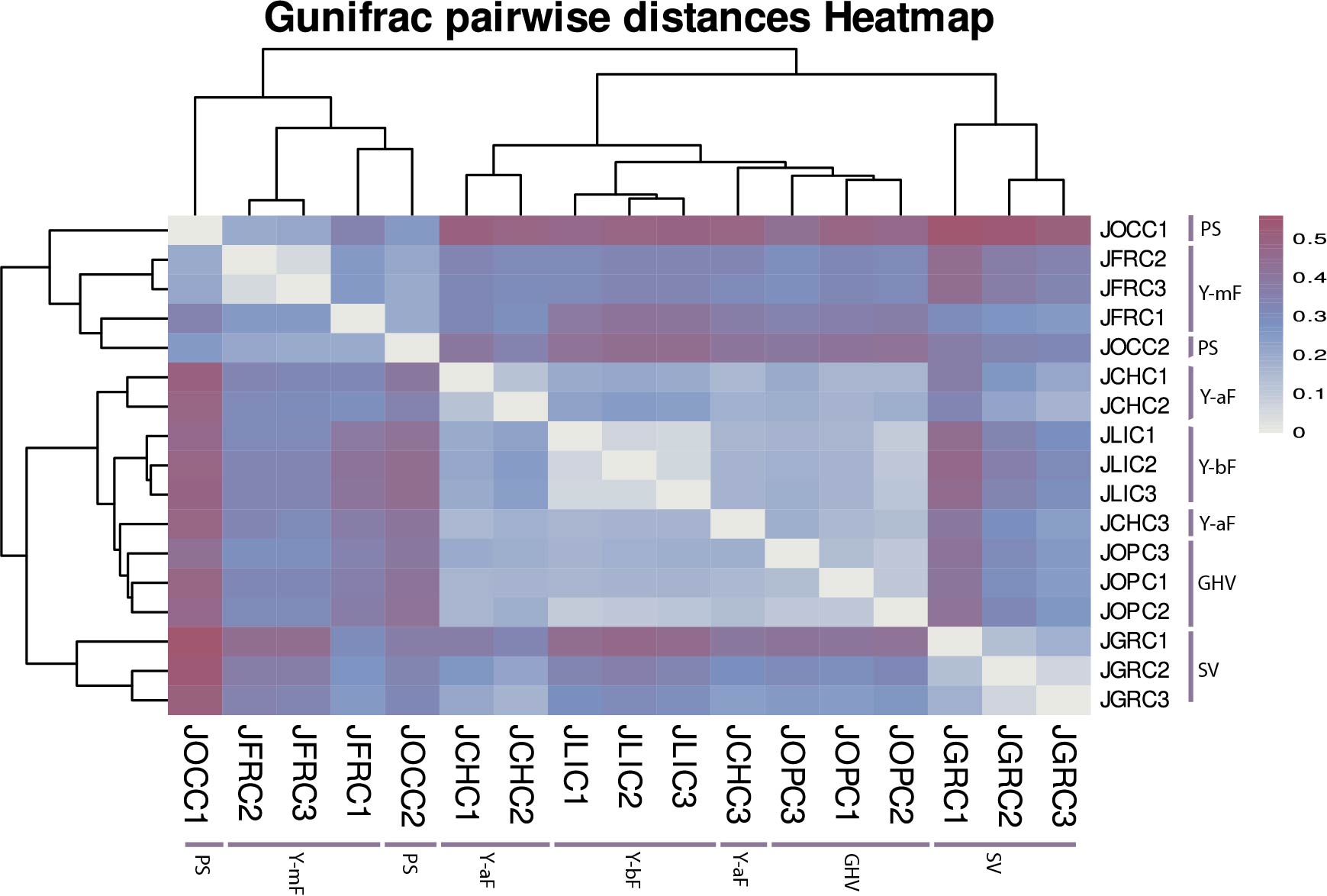


**Figure S9.** Heatmap analysis of bacterial microbiota associated with pollen reserves across six ecosystems, based on GUniFrac pairwise distances. Ecosystems include the Yunga (Pluvial) Altimontane Forest (Y-aF), the Yunga Montane Forest (Y-mF), secondary vegetation (SV) areas, the Yunga Basimontane Forest (Y-bF), the Palm Swamp (PS), and grassland - herbaceous vegetation (GHV) areas.


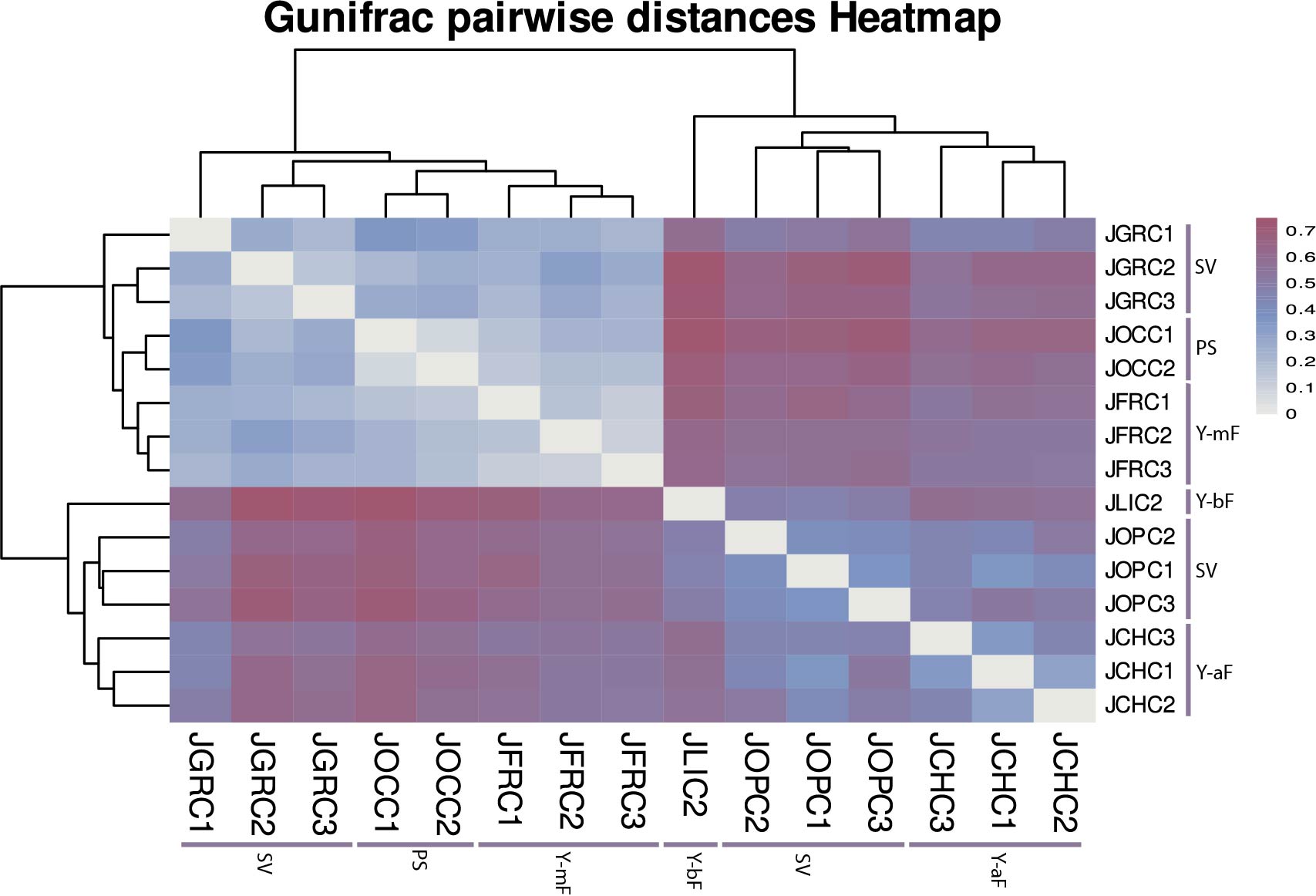


**Figure S10.** Heatmap analysis of fungal microbiota associated with pollen reserves across six ecosystems, based on GUniFrac pairwise distances. Ecosystems include the Yunga (Pluvial) Altimontane Forest (Y-aF), the Yunga Montane Forest (Y-mF), secondary vegetation (SV) areas, the Yunga Basimontane Forest (Y-bF), the Palm Swamp (PS), and grassland - herbaceous vegetation (GHV) areas.


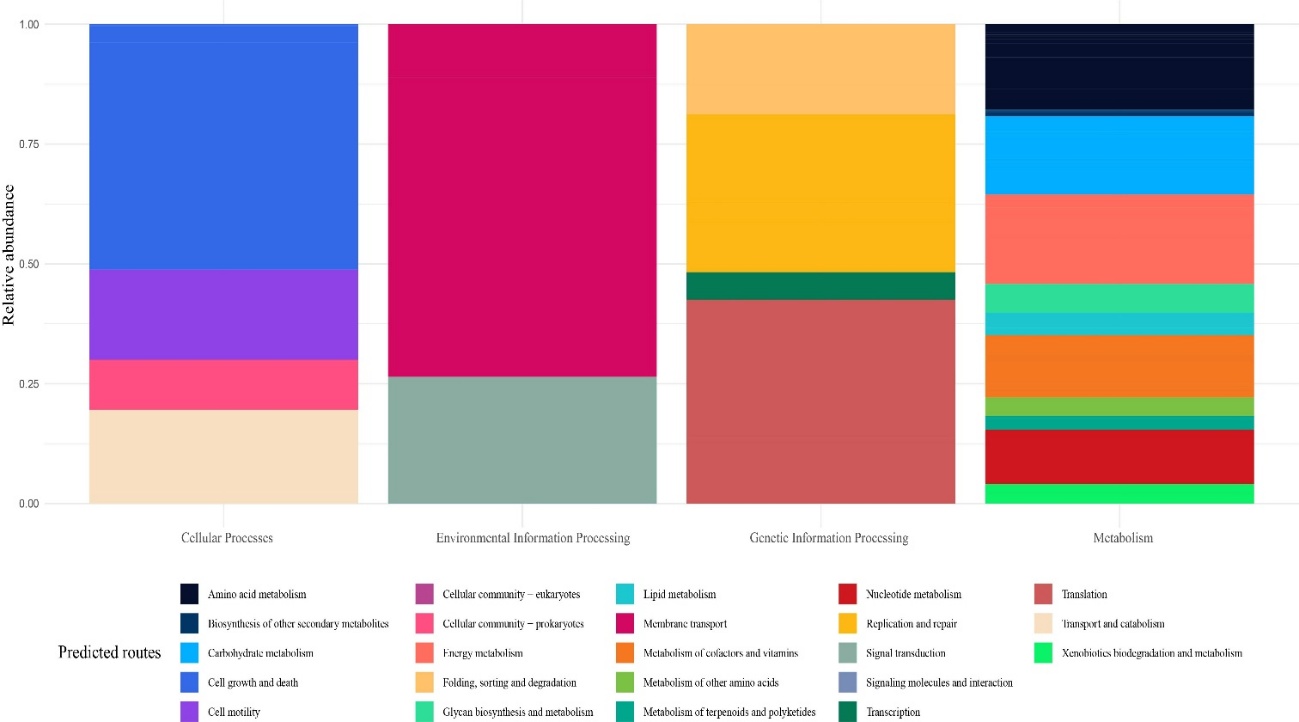


**Figure S11.** Pathways related to the biological functions and subfunctions of the bacterial microbiota associated with bee pollen reserves across six ecosystems in the Amazonas region.
