# Supplementary material for: Hidden Microbiota Inhabiting in Pollen Reserves of Honey Bee (Apis mellifera) From Amazonas Region Revealed by DNA Metabarcoding
Source: Environ Microbiol Rep. 2026 Jul 22;18(4):e70392. doi: 10.1111/1758-2229.70392 (PMC13392498; doi:10.1111/1758-2229.70392)
Supplement: Supplementary file 2 — Table S1: Coordinates and locations of the apiaries evaluated in this study. Table S2: Read quality processing results for the microbiota associated with pollen reserves. Table S3: Total taxonomic composition at the phylum level of bacteria associated with pollen reserves in the Amazonas region. Table S4: Taxonomic composition at the phylum level of bacteria associated with pollen reserves in the six ecosystems in the Amazonas region. Table S5: Total taxonomic composition at the family level of bacteria associated with pollen reserves in the Amazonas region. Table S6: Taxonomic composition at the family level of bacteria associated with pollen reserves in the six ecosystems in the Amazonas region. Table S7: Total taxonomic composition at the genus level of bacteria associated with pollen reserves in the Amazonas region. Table S8: Taxonomic composition at the genus level of bacteria associated with pollen reserves in the six ecosystems in the Amazonas region. Table S9: Total taxonomic composition at the phylum level of fungi associated with pollen reserves in the Amazonas region. Table S10: Taxonomic composition at the phylum level of fungi associated with pollen reserves in the six ecosystems in the Amazonas region. Table S11: Total taxonomic composition at the family level of fungi associated with pollen reserves in the Amazonas region. Table S12: Taxonomic composition at the family level of fungi associated with pollen reserves in the six ecosystems in the Amazonas region. Table S13: Total taxonomic composition at the genus level of fungi associated with pollen reserves in the Amazonas region. Table S14: Taxonomic composition at the genus level of fungi associated with pollen reserves in the six ecosystems in the Amazonas region. Table S15: Alpha diversity indices of bacteria associated with pollen reserves across six ecosystems in the Amazonas region. Table S16: Alpha diversity indices of fungi associated with pollen reserves across six ecosystems in the Amazonas [file EMI4-18-e70392-s001.docx]

**Supplemental Tables**

**Table S1.** Coordinates and locations of the apiaries evaluated in this study.

| **Province** | **District** | **Apiary name** | **Collection code** | **Coordinates** | | | **Ecosystem** | |
| --- | --- | --- | --- | --- | --- | --- | --- | --- |
|  |  |  |  | **Latitude** | **Longitude** | |  |  |
| Chachapoyas | Taquia | Opelel | JOPC1 | -6.260574167 | -77.79891028 | (GHV) | |  |
|  |  |  | JOPC2 |  |  |  |  |  |
|  |  |  | JOPC3 |  |  |  |  |  |
|  | Chiliquin | Chiliquin | JCHC1 | -6.084838333 | -77.753459 | (Y-aF) | |  |
|  |  |  | JCHC2 |  |  |  |  |  |
|  |  |  | JCHC3 |  |  |  |  |  |
|  | Molinompama | Ocol | JOCC1 | -6.0258291944 | -77.565399 | (PS) | |  |
|  |  |  | JOCC2 |  |  |  |  |  |
|  |  |  | JOCC3 |  |  |  |  |  |
| Luya | Camporredondo | Guerrero | JGRC1 | -6.19957 | -78.32008167 | (SV) | |  |
|  |  |  | JGRC2 |  |  |  |  |  |
|  |  |  | JGRC3 |  |  |  |  |  |
| Mendoza | Cochamal | Franco | JFRC1 | -6.39691361 | -77.57709992 | (Y-mF) | |  |
|  |  |  | JFRC2 |  |  |  |  |  |
|  |  |  | JFRC3 |  |  |  |  |  |
| Utcubamba | Cajaruro | Lluhuana | JLIC1 | -5.674914935 | -78.39783031 | (Y-bF) | |  |

**Table S2.** Read quality processing results for the microbiota associated with pollen reserves.

| **Marker** | **Bioproject** | **Biosample** | **Sequencing code** | **Input** | **Filtered** | **DadaF** | **DadaR** | **Merged** | **Nonchim** |
| --- | --- | --- | --- | --- | --- | --- | --- | --- | --- |
| 16S rRNA | PRJNA1415230 | SAMN54926601 | JCHC1_1 | 98069 | 76208 | 75215 | 75376 | 70187 | 33612 |
|  | PRJNA1415230 | SAMN54926602 | JCHC2_1 | 89838 | 70375 | 69343 | 69409 | 64259 | 30053 |
|  | PRJNA1415230 | SAMN54926603 | JCHC3_1 | 104278 | 80319 | 79259 | 79489 | 75664 | 31418 |
|  | PRJNA1415230 | SAMN54926604 | JFRC1_1 | 88541 | 67908 | 67506 | 67586 | 65746 | 35382 |
|  | PRJNA1415230 | SAMN54926605 | JFRC2_1 | 113540 | 88888 | 88631 | 88426 | 86917 | 44388 |
|  | PRJNA1415230 | SAMN54926606 | JFRC3_1 | 97822 | 74400 | 73664 | 73797 | 68495 | 28406 |
|  | PRJNA1415230 | SAMN54926607 | JGRC1_1 | 80930 | 58653 | 58331 | 58345 | 57099 | 29464 |
|  | PRJNA1415230 | SAMN54926608 | JGRC2_1 | 91638 | 68157 | 67683 | 67747 | 65761 | 34998 |
|  | PRJNA1415230 | SAMN54926609 | JGRC3_1 | 85250 | 67652 | 67145 | 67292 | 65076 | 36046 |
|  | PRJNA1415230 | SAMN54926610 | JLIC1_1 | 86217 | 69943 | 69396 | 69266 | 67353 | 40247 |
|  | PRJNA1415230 | SAMN54926611 | JLIC2_1 | 93683 | 73567 | 72992 | 72983 | 70058 | 27049 |
|  | PRJNA1415230 | SAMN54926612 | JLIC3_1 | 109240 | 87743 | 87359 | 87354 | 86174 | 44023 |
|  | PRJNA1415230 | SAMN54926613 | JOCC1_1 | 81331 | 61957 | 61593 | 61483 | 60207 | 30814 |
|  | PRJNA1415230 | SAMN54926614 | JOCC2_1 | 90853 | 69741 | 69347 | 69324 | 68205 | 34033 |
|  | PRJNA1415230 | SAMN54926615 | JOPC1_1 | 86699 | 69075 | 68072 | 68357 | 63383 | 28635 |
|  | PRJNA1415230 | SAMN54926616 | JOPC2_1 | 93392 | 73905 | 72976 | 73153 | 68625 | 33805 |
|  | PRJNA1415230 | SAMN54926617 | JOPC3_1 | 91443 | 71792 | 70921 | 71134 | 67404 | 34076 |
| ITS | PRJNA1415238 | SAMN54927062 | JCHC1_3 | 142497 | 68783 | 68491 | 68544 | 533 | 493 |
|  | PRJNA1415238 | SAMN54927063 | JCHC2_3 | 125408 | 59496 | 59297 | 59315 | 335 | 314 |
|  | PRJNA1415238 | SAMN54927064 | JCHC3_3 | 143448 | 63225 | 63018 | 62933 | 276 | 220 |
|  | PRJNA1415238 | SAMN54927065 | JFRC1_3 | 142510 | 79353 | 79178 | 79099 | 553 | 530 |
|  | PRJNA1415238 | SAMN54927066 | JFRC2_3 | 155629 | 79408 | 79261 | 79224 | 1859 | 1768 |
|  | PRJNA1415238 | SAMN54927067 | JFRC3_3 | 121220 | 61208 | 61084 | 60964 | 391 | 334 |
|  | PRJNA1415238 | SAMN54927068 | JGRC1_3 | 123248 | 70406 | 70344 | 70234 | 1681 | 1630 |
|  | PRJNA1415238 | SAMN54927069 | JGRC2_3 | 140447 | 72304 | 72190 | 72215 | 556 | 528 |
|  | PRJNA1415238 | SAMN54927070 | JGRC3_3 | 132057 | 69018 | 68887 | 68877 | 453 | 438 |
|  | PRJNA1415238 | SAMN54927071 | JLIC2_3 | 131755 | 67325 | 67120 | 67064 | 335 | 330 |
|  | PRJNA1415238 | SAMN54927072 | JOCC1_3 | 111956 | 52636 | 52525 | 52557 | 503 | 500 |
|  | PRJNA1415238 | SAMN54927073 | JOCC2_3 | 115628 | 64560 | 64508 | 64530 | 663 | 650 |
|  | PRJNA1415238 | SAMN54927074 | JOPC1_3 | 164332 | 72545 | 72256 | 72196 | 406 | 367 |
|  | PRJNA1415238 | SAMN54927075 | JOPC2_3 | 158047 | 80651 | 80176 | 80382 | 487 | 402 |
|  | PRJNA1415238 | SAMN54927076 | JOPC3_3 | 129638 | 62304 | 62066 | 62153 | 436 | 391 |

**Table S3.** Total taxonomic composition at the phylum level of bacteria associated with pollen reserves in the Amazonas region.

| **Phylum** | **Total abundance (ASV)** | **Percentage (%)** |
| --- | --- | --- |
| Firmicutes | 1.424.758 | 83.809 |
| Proteobacteria | 252.289 | 14.841 |
| Actinobacteriota | 12.411 | 0.73 |
| Myxococcota | 6.287 | 0.37 |
| Acidobacteriota | 2.111 | 0.124 |
| Bacteroidota | 1.532 | 0.09 |
| Gemmatimonadota | 0.168 | 0.01 |
| Cyanobacteria | 0.158 | 0.009 |
| Bdellovibrionota | 0.144 | 0.008 |
| Verrucomicrobiota | 0.084 | 0.005 |
| Deinococcota | 0.057 | 0.003 |

**Table S4.** Taxonomic composition at the phylum level of bacteria associated with pollen reserves in the six ecosystems in the Amazonas region.

| **Ecosystem** | **Phylum** | **Total abundance (ASV)** | **Percentage (%)** |
| --- | --- | --- | --- |
| Yunga (Pluvial) Altimontane Forest (Y-aF) | Firmicutes | 279.62 | 93.21 |
|  | Proteobacteria | 19.81 | 6.6 |
|  | Myxococcota | 0.21 | 0.07 |
|  | Cyanobacteria | 0.16 | 0.05 |
|  | Bacteroidota | 0.12 | 0.04 |
|  | Actinobacteriota | 0.07 | 0.02 |
| Grassland - Herbaceous Vegetation (GHV) | Firmicutes | 269.32 | 89.77 |
|  | Proteobacteria | 30.53 | 10.18 |
|  | Bacteroidota | 0.15 | 0.05 |
| Palm Swamp (PS) | Proteobacteria | 111.2 | 55.6 |
|  | Firmicutes | 76.12 | 38.06 |
|  | Actinobacteriota | 6.83 | 3.42 |
|  | Myxococcota | 5.85 | 2.92 |
| Yunga Montane Forest (Y-mF) | Firmicutes | 239.59 | 79.86 |
|  | Proteobacteria | 56.43 | 18.81 |
|  | Actinobacteriota | 2.45 | 0.82 |
|  | Acidobacteriota | 1.29 | 0.43 |
|  | Myxococcota | 0.16 | 0.05 |
|  | Bacteroidota | 0.09 | 0.03 |
| Secondary Vegetation (SV) | Firmicutes | 279.08 | 93.03 |
|  | Proteobacteria | 19.67 | 6.56 |
|  | Actinobacteriota | 0.69 | 0.23 |
|  | Acidobacteriota | 0.42 | 0.14 |
|  | Bdellovibrionota | 0.14 | 0.05 |
| Yunga Basimontane Forest (Y-bF) | Firmicutes | 281.02 | 93.67 |
|  | Proteobacteria | 14.65 | 4.88 |
|  | Actinobacteriota | 2.38 | 0.79 |
|  | Bacteroidota | 1.17 | 0.39 |
|  | Acidobacteriota | 0.4 | 0.13 |
|  | Gemmatimonadota | 0.17 | 0.06 |
|  | Verrucomicrobiota | 0.08 | 0.03 |
|  | Deinococcota | 0.06 | 0.02 |
|  | Myxococcota | 0.07 | 0.02 |

**Table S5.** Total taxonomic composition at the family level of bacteria associated with pollen reserves in the Amazonas region.

| **Family** | **Total abundance (ASV)** | **Percentage (%)** |
| --- | --- | --- |
| *Lactobacillaceae* | 1400.41 | 82.377 |
| *Moraxellaceae* | 43.357 | 2.55 |
| *Beijerinckiaceae* | 40.669 | 2.392 |
| *Erwiniaceae* | 37.59 | 2.211 |
| *Morganellaceae* | 34.523 | 2.031 |
| *Enterobacteriaceae* | 32.07 | 1.886 |
| *Pseudomonadaceae* | 23.057 | 1.356 |
| *Leuconostocaceae* | 10.188 | 0.599 |
| *Orbaceae* | 8.09 | 0.476 |
| *Myxococcaceae* | 6.132 | 0.361 |
| *Sphingomonadaceae* | 6.009 | 0.353 |
| *Acetobacteraceae* | 5.904 | 0.347 |
| *Yersiniaceae* | 3.905 | 0.23 |
| *Oxalobacteraceae* | 2.831 | 0.167 |
| *Streptomycetaceae* | 2.524 | 0.148 |
| *Halomonadaceae* | 2.356 | 0.139 |
| *Staphylococcaceae* | 2.089 | 0.123 |
| *Rhizobiaceae* | 1.324 | 0.078 |
| *Tsukamurellaceae* | 1.009 | 0.059 |
| *Comamonadaceae* | 0.699 | 0.041 |
| *Bacillaceae* | 0.611 | 0.036 |
| *Entomoplasmataceae* | 0.444 | 0.026 |
| *Flavobacteriaceae* | 0.447 | 0.026 |
| *Sphingobacteriaceae* | 0.409 | 0.024 |
| *Solibacteraceae* | 0.394 | 0.023 |
| *Xanthomonadaceae* | 0.37 | 0.022 |
| *Pseudonocardiaceae* | 0.355 | 0.021 |
| *Rhodanobacteraceae* | 0.292 | 0.017 |
| *Aeromonadaceae* | 0.312 | 0.018 |
| *Weeksellaceae* | 0.292 | 0.017 |
| *Hyphomicrobiaceae* | 0.234 | 0.014 |
| *Xanthobacteraceae* | 0.239 | 0.014 |
| *Intrasporangiaceae* | 0.187 | 0.011 |
| *Micromonosporaceae* | 0.175 | 0.01 |

**Table S6.** Taxonomic composition at the family level of bacteria associated with pollen reserves in the six ecosystems in the Amazonas region.

| **Ecosystem** | **Family** | **Total abundance (ASV)** | **Percentage (%)** |
| --- | --- | --- | --- |
| Yunga (Pluvial) Altimontane Forest (Y-aF) | *Lactobacillaceae* | 278.56 | 92.85 |
|  | *Moraxellaceae* | 9.54 | 3.18 |
|  | *Pseudomonadaceae* | 3.04 | 1.01 |
|  | *Erwiniaceae* | 1.72 | 0.57 |
|  | *Acetobacteraceae* | 1.37 | 0.46 |
|  | *Morganellaceae* | 1.07 | 0.36 |
|  | *Beijerinckiaceae* | 0.88 | 0.29 |
|  | *Yersiniaceae* | 0.87 | 0.29 |
|  | *Rhodanobacteraceae* | 0.44 | 0.15 |
|  | *Entomoplasmataceae* | 0.42 | 0.14 |
|  | *Rhizobiaceae* | 0.33 | 0.11 |
|  | *Enterococcaceae* | 0.27 | 0.09 |
|  | *Myxococcaceae* | 0.21 | 0.07 |
|  | *Staphylococcaceae* | 0.22 | 0.07 |
|  | *Bacillaceae* | 0.15 | 0.05 |
|  | *Halomonadaceae* | 0.16 | 0.05 |
|  | *Sphingomonadaceae* | 0.16 | 0.05 |
|  | *Oxalobacteraceae* | 0.12 | 0.04 |
|  | *Sphingobacteriaceae* | 0.12 | 0.04 |
|  | *Xanthomonadaceae* | 0.12 | 0.04 |
| Grassland - Herbaceous Vegetation (GHV) | *Lactobacillaceae* | 268.84 | 89.62 |
|  | *Erwiniaceae* | 11.98 | 3.99 |
|  | *Moraxellaceae* | 8.78 | 2.93 |
|  | *Pseudomonadaceae* | 4.11 | 1.37 |
|  | *Orbaceae* | 1.67 | 0.56 |
|  | *Acetobacteraceae* | 1.49 | 0.5 |
|  | *Yersiniaceae* | 1.18 | 0.39 |
|  | *Halomonadaceae* | 0.59 | 0.2 |
|  | *Rhodanobacteraceae* | 0.5 | 0.17 |
|  | *Bacillaceae* | 0.14 | 0.05 |
|  | *Weeksellaceae* | 0.15 | 0.05 |
|  | *Beijerinckiaceae* | 0.12 | 0.04 |
|  | *Rhizobiaceae* | 0.07 | 0.02 |
|  | *Entomoplasmataceae* | 0.02 | 0.01 |
| Palm Swamp (PS) | *Lactobacillaceae* | 59.5 | 29.75 |
|  | *Beijerinckiaceae* | 30.51 | 15.26 |
|  | *Enterobacteriaceae* | 21.97 | 10.99 |
|  | *Erwiniaceae* | 14.36 | 7.18 |
|  | *Morganellaceae* | 11.25 | 5.63 |
|  | *Pseudomonadaceae* | 10.24 | 5.12 |
|  | *Leuconostocaceae* | 7.66 | 3.83 |
|  | *Rhodanobacteraceae* | 6.35 | 3.18 |
|  | *Moraxellaceae* | 6.21 | 3.11 |
|  | *Myxococcaceae* | 5.85 | 2.93 |
|  | *Orbaceae* | 4.9 | 2.45 |
|  | *Sphingomonadaceae* | 3.92 | 1.96 |
|  | *Halomonadaceae* | 1.47 | 0.74 |
| Yunga Montane Forest (Y-mF) | *Lactobacillaceae* | 237.68 | 79.23 |
|  | *Moraxellaceae* | 17.34 | 5.78 |
|  | *Morganellaceae* | 14.86 | 4.95 |
|  | *Erwiniaceae* | 8.13 | 2.71 |
|  | *Beijerinckiaceae* | 5.37 | 1.79 |
|  | *Enterobacteriaceae* | 4.18 | 1.39 |
|  | *Streptomycetaceae* | 2.36 | 0.79 |
|  | *Oxalobacteraceae* | 2.32 | 0.77 |
|  | *Pseudomonadaceae* | 2.28 | 0.76 |
|  | *Staphylococcaceae* | 1.86 | 0.62 |
|  | *Orbaceae* | 1.33 | 0.44 |
|  | *Sphingomonadaceae* | 0.33 | 0.11 |
|  | *Rhizobiaceae* | 0.29 | 0.1 |
|  | *Weeksellaceae* | 0.09 | 0.03 |
| Secondary Vegetation (SV) | *Lactobacillaceae* | 276 | 92 |
|  | *Morganellaceae* | 6.05 | 2.02 |
|  | *Enterobacteriaceae* | 4.04 | 1.35 |
|  | *Beijerinckiaceae* | 3.36 | 1.12 |
|  | *Leuconostocaceae* | 2.43 | 0.81 |
|  | *Acetobacteraceae* | 2.41 | 0.8 |
|  | *Moraxellaceae* | 1.36 | 0.45 |
|  | *Pseudomonadaceae* | 1.32 | 0.44 |
|  | *Rhizobiaceae* | 0.56 | 0.19 |
|  | *Erwiniaceae* | 0.31 | 0.1 |
|  | *Orbaceae* | 0.14 | 0.05 |
|  | *Pseudonocardiaceae* | 0.13 | 0.04 |
|  | *Bacillaceae* | 0.09 | 0.03 |
| Yunga Basimontane Forest (Y-bF) | *Lactobacillaceae* | 279.82 | 93.28 |
|  | *Pseudomonadaceae* | 2.06 | 0.69 |
|  | *Enterobacteriaceae* | 1.88 | 0.63 |
|  | *Yersiniaceae* | 1.85 | 0.62 |
|  | *Sphingomonadaceae* | 1.6 | 0.53 |
|  | *Morganellaceae* | 1.29 | 0.43 |
|  | *Erwiniaceae* | 1.09 | 0.36 |
|  | *Tsukamurellaceae* | 1.01 | 0.34 |
|  | *Comamonadaceae* | 0.7 | 0.23 |
|  | *Acetobacteraceae* | 0.64 | 0.21 |
|  | *Flavobacteriaceae* | 0.45 | 0.15 |
|  | *Beijerinckiaceae* | 0.42 | 0.14 |
|  | *Oxalobacteraceae* | 0.39 | 0.13 |
|  | *Solibacteraceae* | 0.39 | 0.13 |
|  | *Aeromonadaceae* | 0.31 | 0.1 |
|  | *Sphingobacteriaceae* | 0.29 | 0.1 |
|  | *Bacillaceae* | 0.23 | 0.08 |
|  | *Hyphomicrobiaceae* | 0.23 | 0.08 |
|  | *Pseudonocardiaceae* | 0.23 | 0.08 |
|  | *Xanthobacteraceae* | 0.24 | 0.08 |
|  | *Xanthomonadaceae* | 0.25 | 0.08 |
|  | *Intrasporangiaceae* | 0.19 | 0.06 |
|  | *Micromonosporaceae* | 0.17 | 0.06 |
|  | *Halomonadaceae* | 0.14 | 0.05 |
|  | *Mycobacteriaceae* | 0.14 | 0.05 |
|  | *Streptomycetaceae* | 0.16 | 0.05 |
|  | *Moraxellaceae* | 0.12 | 0.04 |
|  | *Leuconostocaceae* | 0.1 | 0.03 |
|  | *Rhizobiaceae* | 0.08 | 0.03 |
|  | *Myxococcaceae* | 0.07 | 0.02 |
|  | *Orbaceae* | 0.05 | 0.02 |
|  | *Weeksellaceae* | 0.05 | 0.02 |

**Table S7.** Total taxonomic composition at the genus level of bacteria associated with pollen reserves in the Amazonas region.

| **Genus** | **Total abundance (ASV)** | **Percentage (%)** |
| --- | --- | --- |
| *Lactobacillus* | 1400.41 | 82.377 |
| *Acinetobacter* | 36.925 | 2.172 |
| *Arsenophonus* | 34.523 | 2.031 |
| *Escherichia-Shigella* | 30.583 | 1.799 |
| *1174-901-12* | 23.841 | 1.402 |
| *Pseudomonas* | 23.057 | 1.356 |
| *Rosenbergiella* | 18.324 | 1.078 |
| *Methylobacterium-Methylorubrum* | 16.733 | 0.984 |
| *Phaseolibacter* | 13.71 | 0.806 |
| *Fructobacillus* | 10.091 | 0.594 |
| *Luteibacter* | 7.294 | 0.429 |
| *Alkanindiges* | 6.432 | 0.378 |
| *Gilliamella* | 6.423 | 0.378 |
| *Sphingomonas* | 5.539 | 0.326 |
| *Erwinia* | 5.071 | 0.298 |
| *Bombella* | 4.651 | 0.274 |
| *Serratia* | 3.417 | 0.201 |
| *Streptomyces* | 2.524 | 0.148 |
| *Staphylococcus* | 2.089 | 0.123 |
| *Frischella* | 1.667 | 0.098 |
| *Tsukamurella* | 1.009 | 0.059 |
| *Lelliottia* | 0.921 | 0.054 |
| *Acidibacter* | 0.713 | 0.042 |
| *Comamonas* | 0.562 | 0.033 |
| *Bacillus* | 0.516 | 0.03 |
| *Aureimonas* | 0.476 | 0.028 |
| *Flavobacterium* | 0.447 | 0.026 |
| *Mesoplasma* | 0.444 | 0.026 |
| *Zymobacter* | 0.411 | 0.024 |
| *Candidatus Solibacter* | 0.394 | 0.023 |
| *Sphingobium* | 0.356 | 0.021 |
| *Aeromonas* | 0.312 | 0.018 |
| *Pedomicrobium* | 0.234 | 0.014 |
| *Pseudolabrys* | 0.239 | 0.014 |
| *Tatumella* | 0.2 | 0.012 |

**Table S8.** Taxonomic composition at the genus level of bacteria associated with pollen reserves in the six ecosystems in the Amazonas region.

| **Ecosystem** | **Genus** | **Total abundance (ASV)** | **Percentage (%)** |
| --- | --- | --- | --- |
| Yunga (Pluvial) Altimontane Forest (Y-aF) | *Lactobacillus* | 278.56 | 92.85 |
|  | *Acinetobacter* | 7.26 | 2.42 |
|  | *Pseudomonas* | 3.04 | 1.01 |
|  | *Alkanindiges* | 2.28 | 0.76 |
|  | *Erwinia* | 1.72 | 0.57 |
|  | *Arsenophonus* | 1.07 | 0.36 |
|  | *Serratia* | 0.87 | 0.29 |
|  | *Bombella* | 0.8 | 0.27 |
|  | *Methylobacterium-Methylorubrum* | 0.63 | 0.21 |
|  | *Luteibacter* | 0.44 | 0.15 |
|  | *Mesoplasma* | 0.42 | 0.14 |
|  | *Aureimonas* | 0.33 | 0.11 |
|  | *1174-901-12* | 0.25 | 0.08 |
|  | *Staphylococcus* | 0.22 | 0.07 |
|  | *Bacillus* | 0.15 | 0.05 |
|  | *Sphingomonas* | 0.16 | 0.05 |
|  | *Zymobacter* | 0.16 | 0.05 |
| Grassland - Herbaceous Vegetation (GHV) | *Lactobacillus* | 268.84 | 89.62 |
|  | *Acinetobacter* | 6.15 | 2.05 |
|  | *Rosenbergiella* | 5.93 | 1.98 |
|  | *Pseudomonas* | 4.11 | 1.37 |
|  | *Phaseolibacter* | 2.94 | 0.98 |
|  | *Erwinia* | 2.84 | 0.95 |
|  | *Alkanindiges* | 2.63 | 0.88 |
|  | *Frischella* | 1.67 | 0.56 |
|  | *Bombella* | 1.49 | 0.5 |
|  | *Serratia* | 0.69 | 0.23 |
|  | *Luteibacter* | 0.5 | 0.17 |
|  | *Bacillus* | 0.14 | 0.05 |
|  | *Methylobacterium-Methylorubrum* | 0.12 | 0.04 |
|  | *Entomoplasmataceae* | 0.02 | 0.01 |
|  | *Zymobacter* | 0.12 | 0.04 |
|  | *Aureimonas* | 0.07 | 0.02 |
|  | *Mesoplasma* | 0.02 | 0.01 |
| Palm Swamp (PS) | *Lactobacillus* | 59.5 | 29.75 |
|  | *Escherichia-Shigella* | 21.97 | 10.98 |
|  | *1174-901-12* | 19.42 | 9.71 |
|  | *Arsenophonus* | 11.25 | 5.62 |
|  | *Methylobacterium-Methylorubrum* | 11.09 | 5.54 |
|  | *Phaseolibacter* | 10.46 | 5.23 |
|  | *Pseudomonas* | 10.24 | 5.12 |
|  | *Fructobacillus* | 7.66 | 3.83 |
|  | *Luteibacter* | 6.35 | 3.17 |
|  | *Gilliamella* | 4.9 | 2.45 |
|  | *Rosenbergiella* | 3.91 | 1.96 |
|  | *Sphingomonas* | 3.92 | 1.96 |
| Yunga Montane Forest (Y-mF) | *Lactobacillus* | 237.68 | 79.23 |
|  | *Acinetobacter* | 15.85 | 5.28 |
|  | *Arsenophonus* | 14.86 | 4.95 |
|  | *Rosenbergiella* | 8.13 | 2.71 |
|  | *Methylobacterium-Methylorubrum* | 4.08 | 1.36 |
|  | *Escherichia-Shigella* | 3.93 | 1.31 |
|  | *Streptomyces* | 2.36 | 0.79 |
|  | *Pseudomonas* | 2.28 | 0.76 |
|  | *Staphylococcus* | 1.86 | 0.62 |
|  | *Alkanindiges* | 1.49 | 0.5 |
|  | *Gilliamella* | 1.33 | 0.44 |
|  | *1174-901-12* | 1.29 | 0.43 |
|  | *Sphingomonas* | 0.33 | 0.11 |
| Secondary Vegetation (SV) | *Lactobacillus* | 276 | 92 |
|  | *Arsenophonus* | 6.05 | 2.02 |
|  | *Escherichia-Shigella* | 4.04 | 1.35 |
|  | *1174-901-12* | 2.82 | 0.94 |
|  | *Fructobacillus* | 2.43 | 0.81 |
|  | *Bombella* | 2.06 | 0.69 |
|  | *Acinetobacter* | 1.36 | 0.45 |
|  | *Pseudomonas* | 1.32 | 0.44 |
|  | *Methylobacterium-Methylorubrum* | 0.54 | 0.18 |
|  | *Gilliamella* | 0.14 | 0.05 |
|  | *Bacillus* | 0.09 | 0.03 |
| Yunga Basimontane Forest (Y-bF) | *Lactobacillus* | 279.82 | 93.29 |
|  | *Pseudomonas* | 2.06 | 0.69 |
|  | *Serratia* | 1.85 | 0.62 |
|  | *Arsenophonus* | 1.29 | 0.43 |
|  | *Sphingomonas* | 1.13 | 0.38 |
|  | *Tsukamurella* | 1.01 | 0.34 |
|  | *Lelliottia* | 0.92 | 0.31 |
|  | *Acidibacter* | 0.71 | 0.24 |
|  | *Escherichia-Shigella* | 0.64 | 0.21 |
|  | *Comamonas* | 0.56 | 0.19 |
|  | *Erwinia* | 0.51 | 0.17 |
|  | *Flavobacterium* | 0.45 | 0.15 |
|  | *Candidatus Solibacter* | 0.39 | 0.13 |
|  | *Rosenbergiella* | 0.36 | 0.12 |
|  | *Sphingobium* | 0.36 | 0.12 |
|  | *Aeromonas* | 0.31 | 0.1 |
|  | *Bombella* | 0.3 | 0.1 |
|  | *Methylobacterium-Methylorubrum* | 0.27 | 0.09 |
|  | *Pedomicrobium* | 0.23 | 0.08 |
|  | *Pseudolabrys* | 0.24 | 0.08 |
|  | *Tatumella* | 0.2 | 0.07 |
|  | *Bacillus* | 0.14 | 0.05 |
|  | *Streptomyces* | 0.16 | 0.05 |
|  | *Zymobacter* | 0.14 | 0.05 |

**Table S9.** Total taxonomic composition at the phylum level of fungi associated with pollen reserves in the Amazonas region.

| **Phylum** | **Total abundance (ASV)** | **Percentage (%)** |
| --- | --- | --- |
| Basidiomycota | 1.495.266 | 99.684 |
| Fungi_phy_Incertae_sedis | 2.959 | 0.197 |
| Ascomycota | 1.479 | 0.099 |
| Fungi Kingdom | 0.296 | 0.02 |

**Table S10.** Taxonomic composition at the phylum level of fungi associated with pollen reserves in the six ecosystems in the Amazonas region.

| **Ecosystem** | **Phylum** | **Total abundance (ASV)** | **Percentage (%)** |
| --- | --- | --- | --- |
| Yunga (Pluvial) Altimontane Forest (Y-aF) | p__Basidiomycota | 297.04 | 99.01 |
|  | p__Fungi_phy_Incertae_sedis | 2.96 | 0.99 |
| Grassland - Herbaceous Vegetation (GHV) | p__Basidiomycota | 300 | 100 |
| Palm Swamp (PS) | p__Basidiomycota | 200 | 100 |
| Yunga Montane Forest (Y-mF) | p__Basidiomycota | 300 | 100 |
| Secondary Vegetation (SV) | p__Basidiomycota | 300 | 100 |
| Yunga Basimontane Forest (Y-bF) | p__Basidiomycota | 98.22 | 98.22 |
|  | p__Ascomycota | 1.48 | 1.48 |
|  | k__Fungi Kingdom | 0.3 | 0.3 |

**Table S11.** Total taxonomic composition at the family level of fungi associated with pollen reserves in the Amazonas region.

| **Family** | **Total abundance (ASV)** | **Percentage (%)** |
| --- | --- | --- |
| c__Tremellomycetes Class | 957.988 | 63.866 |
| f__Bulleribasidiaceae | 523.373 | 34.892 |
| f__Holtermanniaceae | 13.905 | 0.927 |
| f__Fungi_fam_Incertae_sedis | 2.959 | 0.197 |
| f__Saccharomycetaceae | 1.479 | 0.099 |
| k__Fungi Kingdom | 0.296 | 0.02 |

**Table S12.** Taxonomic composition at the family level of fungi associated with pollen reserves in the six ecosystems in the Amazonas region.

| **Ecosystem** | **Family** | **Total abundance** | **Percentage** |
| --- | --- | --- | --- |
| Yunga (Pluvial) Altimontane Forest (Y-aF) | f__Bulleribasidiaceae | 236.98 | 78.99 |
|  | c__Tremellomycetes Class | 56.51 | 18.84 |
|  | f__Holtermanniaceae | 3.55 | 1.18 |
|  | f__Fungi_fam_Incertae_sedis | 2.96 | 0.99 |
| Grassland - Herbaceous Vegetation (GHV) | f__Bulleribasidiaceae | 214.79 | 71.6 |
|  | c__Tremellomycetes Class | 74.85 | 24.95 |
|  | f__Holtermanniaceae | 10.36 | 3.45 |
| Palm Swamp (PS) | c__Tremellomycetes Class | 194.38 | 97.19 |
|  | f__Bulleribasidiaceae | 5.62 | 2.81 |
| Yunga Montane Forest (Y-mF) | c__Tremellomycetes Class | 265.98 | 88.66 |
|  | f__Bulleribasidiaceae | 34.02 | 11.34 |
| Secondary Vegetation (SV) | c__Tremellomycetes Class | 275.15 | 91.72 |
|  | f__Bulleribasidiaceae | 24.85 | 8.28 |
| Yunga Basimontane Forest (Y-bF) | c__Tremellomycetes Class | 91.12 | 91.12 |
|  | f__Bulleribasidiaceae | 7.1 | 7.1 |
|  | f__Saccharomycetaceae | 1.48 | 1.48 |
|  | k__Fungi Kingdom | 0.3 | 0.3 |

**Table S13.** Total taxonomic composition at the genus level of fungi associated with pollen reserves in the Amazonas region.

| **Genus** | **Total abundance (ASV)** | **Percentage (%)** |
| --- | --- | --- |
| c__Tremellomycetes Class | 957.988 | 63.866 |
| f__Bulleribasidiaceae Family | 414.793 | 27.653 |
| g__Vishniacozyma | 107.101 | 7.14 |
| g__Holtermannia | 13.905 | 0.927 |
| g__Fungi_gen_Incertae_sedis | 2.959 | 0.197 |
| g__Derxomyces | 1.479 | 0.099 |
| g__Zygotorulaspora | 1.479 | 0.099 |
| k__Fungi Kingdom | 0.296 | 0.02 |

**Table S14.** Taxonomic composition at the genus level of fungi associated with pollen reserves in the six ecosystems in the Amazonas region.

| **Ecosystem** | **Genus** | **Total abundance (ASV)** | **Percentage (%)** |
| --- | --- | --- | --- |
| Yunga (Pluvial) Altimontane Forest (Y-aF) | f__Bulleribasidiaceae Family | 211.83 | 70.61 |
|  | c__Tremellomycetes Class | 56.51 | 18.84 |
|  | *g__Vishniacozyma* | 23.96 | 7.99 |
|  | *g__Holtermannia* | 3.55 | 1.18 |
|  | *g__Fungi_gen_Incertae_sedis* | 2.96 | 0.99 |
|  | *g__Derxomyces* | 1.18 | 0.39 |
| Grassland - Herbaceous Vegetation (GHV) | f__Bulleribasidiaceae Family | 138.76 | 46.25 |
|  | *g__Vishniacozyma* | 75.74 | 25.25 |
|  | c__Tremellomycetes Class | 74.85 | 24.95 |
|  | *g__Holtermannia* | 10.36 | 3.45 |
|  | *g__Derxomyces* | 0.3 | 0.1 |
| Palm Swamp (PS) | c__Tremellomycetes Class | 194.38 | 97.19 |
|  | f__Bulleribasidiaceae Family | 5.62 | 2.81 |
| Yunga Montane Forest (Y-mF) | c__Tremellomycetes Class | 265.98 | 88.66 |
|  | f__Bulleribasidiaceae Family | 26.63 | 8.88 |
|  | *g__Vishniacozyma* | 7.4 | 2.47 |
| Secondary Vegetation (SV) | c__Tremellomycetes Class | 275.15 | 91.72 |
|  | f__Bulleribasidiaceae Family | 24.85 | 8.28 |
| Yunga Basimontane Forest (Y-bF) | c__Tremellomycetes Class | 91.12 | 91.12 |
|  | f__Bulleribasidiaceae Family | 7.1 | 7.1 |
|  | *g__Zygotorulaspora* | 1.48 | 1.48 |
|  | k__Fungi Kingdom | 0.3 | 0.3 |

**Table S15.** Alpha diversity indices of bacteria associated with pollen reserves across six ecosystems in the Amazonas region.

| **Ecosystem** | **Index** | |
| --- | --- | --- |
|  | **Shannon** | **Simpson** |
| Yunga (Pluvial) Altimontane Forest (Y-aF) | 5.3 | 0.993 |
| Grassland - Herbaceous Vegetation (GHV) | 5.2 | 0.988 |
| Secondary Vegetation (SV) | 5.1 | 0.992 |
| Yunga Montane Forest (Y-mF) | 4.9 | 0.990 |
| Yunga Basimontane Forest (Y-bF) | 4.3 | 0.981 |
| Palm Swamp (PS) | 3.7 | 0.970 |

**Table S16.** Alpha diversity indices of fungi associated with pollen reserves across six ecosystems in the Amazonas region.

| **Ecosystem** | **Index** | |
| --- | --- | --- |
|  | **Shannon** | **Simpson** |
| Grassland - Herbaceous Vegetation (GHV) | 2.3 | 0.885 |
| Yunga (Pluvial) Altimontane Forest (Y-aF) | 2.28 | 0.875 |
| Yunga Montane Forest (Y-mF) | 2.1 | 0.84 |
| Yunga Basimontane Forest (Y-bF) | 2.03 | 0.85 |
| Secondary Vegetation (SV) | 2.15 | 0.86 |
| Palm Swamp (PS) | 1.3 | 0.71 |
